# Shisa6 traps AMPA receptors at postsynaptic sites and prevents their desensitization during synaptic activity

**DOI:** 10.1038/ncomms10682

**Published:** 2016-03-02

**Authors:** Remco V. Klaassen, Jasper Stroeder, Françoise Coussen, Anne-Sophie Hafner, Jennifer D. Petersen, Cedric Renancio, Leanne J. M. Schmitz, Elisabeth Normand, Johannes C. Lodder, Diana C. Rotaru, Priyanka Rao-Ruiz, Sabine Spijker, Huibert D. Mansvelder, Daniel Choquet, August B. Smit

**Affiliations:** 1 Department Molecular and Cellular Neurobiology, 1081 HV Amsterdam, The Netherlands; 2 Department Integrative Neurophysiology, Center for Neurogenomics and Cognitive Research, Neuroscience Campus Amsterdam, VU University, De Boelelaan 1085, 1081 HV Amsterdam, The Netherlands; 3 University of Bordeaux, Interdisciplinary Institute for Neuroscience, UMR 5297, F-33000 Bordeaux, France; 4 CNRS, Interdisciplinary Institute for Neuroscience, UMR 5297, F-33000 Bordeaux, France

## Abstract

Trafficking and biophysical properties of AMPA receptors (AMPARs) in the brain depend on interactions with associated proteins. We identify Shisa6, a single transmembrane protein, as a stable and directly interacting *bona fide* AMPAR auxiliary subunit. Shisa6 is enriched at hippocampal postsynaptic membranes and co-localizes with AMPARs. The Shisa6 C-terminus harbours a PDZ domain ligand that binds to PSD-95, constraining mobility of AMPARs in the plasma membrane and confining them to postsynaptic densities. Shisa6 expressed in HEK293 cells alters GluA1- and GluA2-mediated currents by prolonging decay times and decreasing the extent of AMPAR desensitization, while slowing the rate of recovery from desensitization. Using gene deletion, we show that Shisa6 increases rise and decay times of hippocampal CA1 miniature excitatory postsynaptic currents (mEPSCs). Shisa6-containing AMPARs show prominent sustained currents, indicating protection from full desensitization. Accordingly, Shisa6 prevents synaptically trapped AMPARs from depression at high-frequency synaptic transmission.

## Abstract (web summary)

Auxiliary AMPA receptor subunits can affect gating and surface mobility. Here the authors show that Shisa6 traps AMPA receptors at postsynaptic sites via PSD-95, and keeps them in an activated state in the presence of glutamate, preventing full desensitization and consequently synaptic depression.

## Graphical abstract

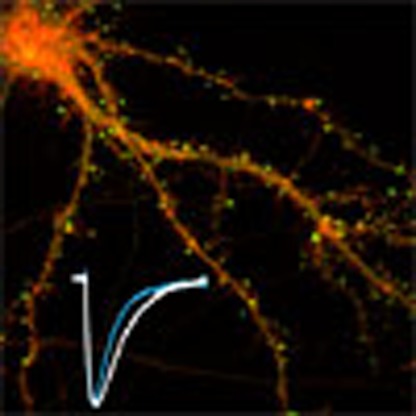

Fast excitatory synaptic transmission in the adult brain is predominantly mediated by AMPA-type glutamate receptors (AMPARs). The strength of glutamatergic transmission can be adjusted in an activity-dependent manner by different mechanisms in pre- and postsynaptic elements^1^,^2^, postsynaptic plasticity being largely determined by regulation of both the number and gating properties of AMPARs^3^,^4^,^5^,^6^,^7^,^8^,^9^. Post-translational modifications and protein interactions enable activity-dependent plasticity underlying learning, memory and synapse turnover^10^,^11^,^12^,^13^. Identification of additional components of native brain-derived AMPAR complexes has revealed a wide variety of mostly transmembrane proteins that directly interact with AMPARs^14^. These proteins can potentially act as auxiliary subunits of AMPARs and affect channel kinetics, trafficking, surface mobility and activity-dependent regulation of these processes. Well-established AMPAR auxiliary subunits include the transmembrane AMPA receptor regulatory proteins (TARPs)^15^,^16^, the Cornichon homologues (CNIH-2 and CNIH-3)^17^ and the recently identified cystine-knot AMPA receptor modulating protein (CKAMP44)^18^,^19^, officially named Shisa9 (ref. 20; Supplementary Fig. 1). Both TARPs and Cornichons decrease deactivation and desensitization rates of the activated AMPAR, and promote synaptic targeting. Overexpression in CA1 of CKAMP44/Shisa9 increases the AMPAR deactivation time constant, slows down recovery from desensitization and decreases AMPAR short-term plasticity^21^. In contrast to CKAMP44/Shisa9 (refs 21, 22), which is expressed most prominently in the hippocampus dentate gyrus, Shisa6 is highly expressed throughout the hippocampus, in dentate gyrus as well as CA regions^23^. Although Shisa6 is part of the AMPAR immunoprecipitated complex^14^, it is as yet unknown whether Shisa6 interacts directly with AMPARs, and whether it affects AMPAR channel kinetics and/or surface expression. Here we demonstrate that Shisa6 is an auxiliary subunit of the AMPAR, which traps these receptors at postsynaptic sites through interaction with PSD-95/DLG4. By altering biophysical properties of AMPARs, Shisa6 keeps AMPARs in an activated state in the presence of glutamate, preventing full desensitization and synaptic depression.

## Results

### Shisa6 is expressed at hippocampal synapses

**Figure 1:**
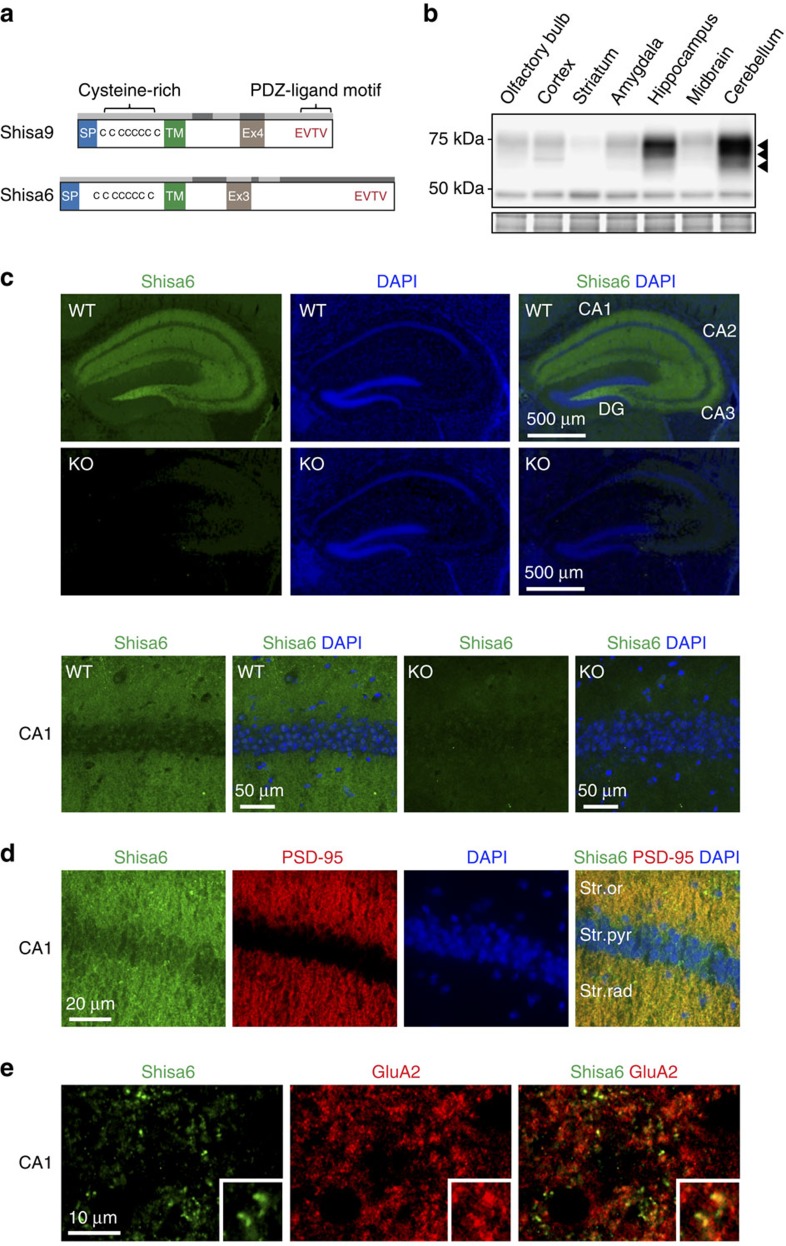
Shisa6 is a type I transmembrane protein enriched in hippocampal dendrites. (**a**) Shisa6 is closely related to the AMPAR auxiliary subunit Shisa9, featuring a signal peptide (SP; 30 amino acids), extracellular domain with conserved cysteine-rich motif, single transmembrane region (TM) and intracellular domain with PDZ-ligand motif (EVTV). Exon 4 (Ex4) is an alternative-splice region in *Shisa9*, whereas this is exon 3 (Ex3; 32 amino acids) in *Shisa6* (Supplementary Fig. 1). The predicted molecular weight of the two mature Shisa6 protein variants is 58.7 and 55.3 kDa, although these have been erroneously assigned a mature mass of 52 kDa previously^14^. The exon structure is indicated above the protein structure by alternating light–dark grey boxes. (**b**) Shisa6 is highly enriched in the hippocampus and cerebellum as measured in crude synaptic membrane fractions. Different molecular weights (∼73, ∼66 and ∼59 kDa; arrow heads) of the Shisa6 protein are apparent. The ∼48-kDa signal is not specific to Shisa6 (Supplementary Fig. 2). Lower panel depicts the loading control, that is, total crude synaptic membrane protein. For complete blots, see Supplementary Fig. 9. (**c**) Immunohistochemistry of WT and *Shisa6* KO brain slices showing Shisa6 (green) expression in the hippocampus (upper panels), and in dendrites of the CA1 (lower panels; zoom-in). DAPI is shown in blue. (**d**) Zoom-in of CA1 staining in WT brain slices shows enrichment of Shisa6 (green) in dendrites, where it co-localizes with PSD-95 (red); DAPI is shown in blue. Cell layers of the CA1 are shown (Str.or, stratum oriens; Str.pyr, stratum pyramidale; Str.rad, stratum radiatum). (**e**) Zoom-in of CA1 dendrites shows Shisa6 (green) co-localization with GluA2 (red). Inset shows a twofold enlargement.

Shisa6 shares high sequence identity with the established AMPAR-associated protein CKAMP44/Shisa9 (Fig. 1a), and features the Shisa family's signature cysteine-rich motif, a single-pass transmembrane region and a type-II PDZ-ligand motif (EVTV) at the C-terminal tail of the intracellular domain (Supplementary Fig. 1). Real-time PCR indicated abundant expression of the *Shisa6* gene in the brain (Supplementary Fig. 1). *In situ* hybridization analysis^23^ revealed expression in the cerebellar Purkinje layer and the hippocampal CA1–3 and dentate gyrus regions, with the latter both in the polymorphic (hilus) and granular layer. In the hippocampus, we detected a single *Shisa6* transcript form containing the alternatively spliced exon 3 (Supplementary Fig. 1). A *Shisa6* knockout (KO) mouse was generated (Supplementary Fig. 2). Immunoblotting with a Shisa6-specific antibody (Supplementary Fig. 2) showed highly enriched expression of Shisa6 in the hippocampus and cerebellum (Fig. 1b). The Shisa6 protein in hippocampus was found to be glycosylated. Treatment with PNGase-F reduced the observed molecular weight of Shisa6 from ∼73 to ∼59 kDa (Supplementary Fig. 2), with the latter being in agreement with the 58.7 kDa predicted for the mature form of exon3-containing Shisa6. Cellular immunostaining comparing wild-type (WT) and KO mice shows dendritic staining within the hippocampus (Fig. 1c). In CA1, CA3 and the polymorphic dentate gyrus, Shisa6 is clearly expressed in the dendritic regions, such as CA1 stratum oriens and stratum radiatum (Fig. 1c,d), CA3 stratum oriens and stratum lucidum, and the dentate gyrus polymorphic layer (Supplementary Fig. 3), the latter of which is known to express AMPARs as well^24^. Dendritic staining can be observed to a lesser extent in the dendrites of the dentate gyrus molecular layer (Supplementary Fig. 3). Shisa6 co-localizes with PSD-95, a scaffolding protein localized to the PSD (postsynaptic density; Fig. 1d), as well as with GluA2 (Fig. 1e) in the CA1 region.

**Figure 2:**
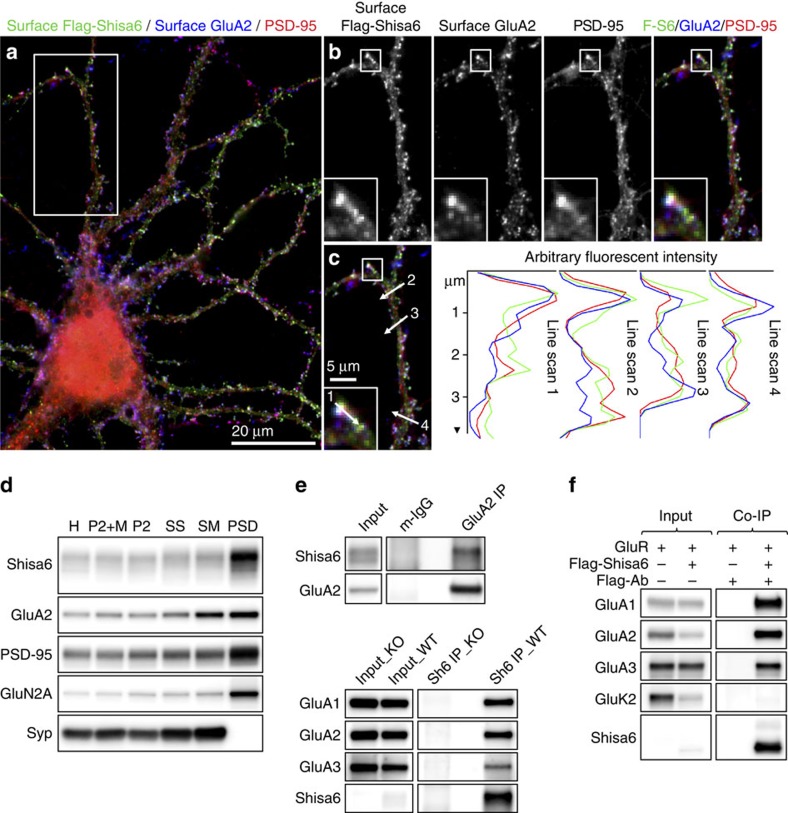
Shisa6 co-localizes with AMPARs and PSD-95 at postsynaptic sites of hippocampal neurons. (**a**) Triple-immunofluorescence staining of a cultured Shisa6 KO neuron (DIV16), expressing inducible Flag-tagged Shisa6 for 18 h, for surface expressed Flag-Shisa6 (green), endogenous surface GluA2 (blue) and endogenous PSD-95 (red) shown as a three-channel overlay. (**b**) Single-channel images and colour overlay of the dendrite region boxed in **a**. An individual synaptic spine (boxed area) is enlarged and is shown (bottom left inset). (**c**) Arrows on overlay image of dendrite segment shown in **b** (left) represent locations of four line scans used to derive graphs shown (right) and illustrate the co-enrichment of Flag-Shisa6, GluA2 and PSD-95 immunofluorescence intensities at synaptic sites. (**d**) Biochemical fractionation (homogenate (H), crude synaptic membranes (P2; with and without microsomes (M)), synaptosomes (SS), synaptic membranes (SM) and PSD fraction (Triton-X100 insoluble fraction) of mature mouse hippocampus reveals an enrichment of Shisa6 in the PSD together with GluA2, GluN2A (NR2A), PSD-95, and distinct from the presynaptic marker synaptophysin (Syp). (**e**) Immunoblot analysis of native hippocampal immunoprecipitated GluA2 complexes reveals the co-precipitation of Shisa6 (upper panel). Immunoblot analysis of immunoprecipitated Shisa6 complexes confirms the interaction with GluA2, and identifies GluA1 and GluA3 as additional interaction partners (lower panel). No signal was obtained in the *Shisa6* KO. The input controls represent 3% of the total lysate. (**f**) Flag-Shisa6 (∼61 kDa) binds directly to homomeric GluA1, GluA2 and GluA3 receptors, while having minimal affinity for GluK2, as shown by co-precipitation from HEK293 cells, using a Flag antibody. The input controls represent 2% of the total lysate. For complete blots, in addition to those with higher exposure, see Supplementary Fig. 9.

The subcellular distribution of surface Shisa6 was further explored by immunofluorescence staining of inducible Flag-Shisa6 expression in *Shisa6* KO primary hippocampal neurons at 16 days *in vitro* (DIV16), as our antibody to native Shisa6 did not label primary neuronal cultures with sufficient specificity. After live staining for Flag-Shisa6 and endogenous GluA2, neurons were permeabilized with Triton-X100 and stained for endogenous synaptic PSD-95 (Fig. 2a,b). Flag-Shisa6 staining is highly enriched at dendritic spines and synaptic sites identified by PSD-95 staining, along with GluA2 (Fig. 2b, inset). A moderate level of extrasynaptic staining was also observed for Shisa6, GluA2 and PSD-95, as evidenced by line scans drawn along spine-like dendritic protrusions and dendrites (Fig. 2c). Altogether, Flag-Shisa6, GluA2 and PSD-95 display a high level of co-localization and enrichment at postsynapses. In agreement, subcellular fractionation of hippocampal proteins revealed that Shisa6 is highly enriched in the Triton-X100-insoluble PSD fraction, in which it co-purified with PSD-95, GluA2 and GluN2A (Fig. 2d).

### Shisa6 interacts with AMPARs

Next, we addressed whether Shisa6 is an AMPAR-interacting protein. First, we investigated the presence of Shisa6 in native hippocampal AMPAR protein complexes, by immunoprecipitation from the n-Dodecyl β-D-maltoside-extracted crude synaptic membrane fraction using an antibody specific for AMPAR subunit GluA2. Indeed, Shisa6 is contained within GluA2 complexes, and absent in the IgG control (Fig. 2e). Immunoprecipitation of native Shisa6 protein complexes from hippocampus confirmed the stable association between Shisa6 and GluA2 (Fig. 2e). In addition, it identified GluA1 and GluA3 subunits as part of the Shisa6 protein complex (Fig. 2e and Supplementary Table 1).

We then investigated whether the interaction between Shisa6 and the AMPAR is subunit specific by co-expression of Shisa6 with AMPAR subunits GluA1, GluA2, GluA3 and kainate receptor subunit GluK2 in heterologous HEK293 cells. GluA1, GluA2, GluA3 and GluK2 were each expressed individually as monomeric receptors in the presence or absence of Flag-Shisa6. Immunoblot analysis revealed that GluA1, GluA2 and GluA3 were co-immunoprecipitated with Flag-Shisa6 (Fig. 2f). GluK2 was not pulled-down with Flag-Shisa6. In conclusion, Shisa6 binds to AMPAR subunits GluA1–3, with similar preference for each of these subunits, but not to kainate receptor subunits. Finally, immunoprecipitation of native Shisa6 complexes from hippocampal synaptic membranes, followed by mass spectrometry, validated our previous findings, and in addition, identified the proteins TARP-gamma8, PRRT1, SAP102 and PSD-95 from the established AMPAR-interactome^14^ as associated with Shisa6 (Supplementary Table 1). However, these interactors were observed with modest spectral counts and found to be absent in Shisa6-GluA1/2 complexes derived from HEK293 cells, suggesting that these proteins are not required for the interaction between Shisa6 and the AMPAR.

### Native Shisa6 interacts directly with PSD-95

**Figure 3:**
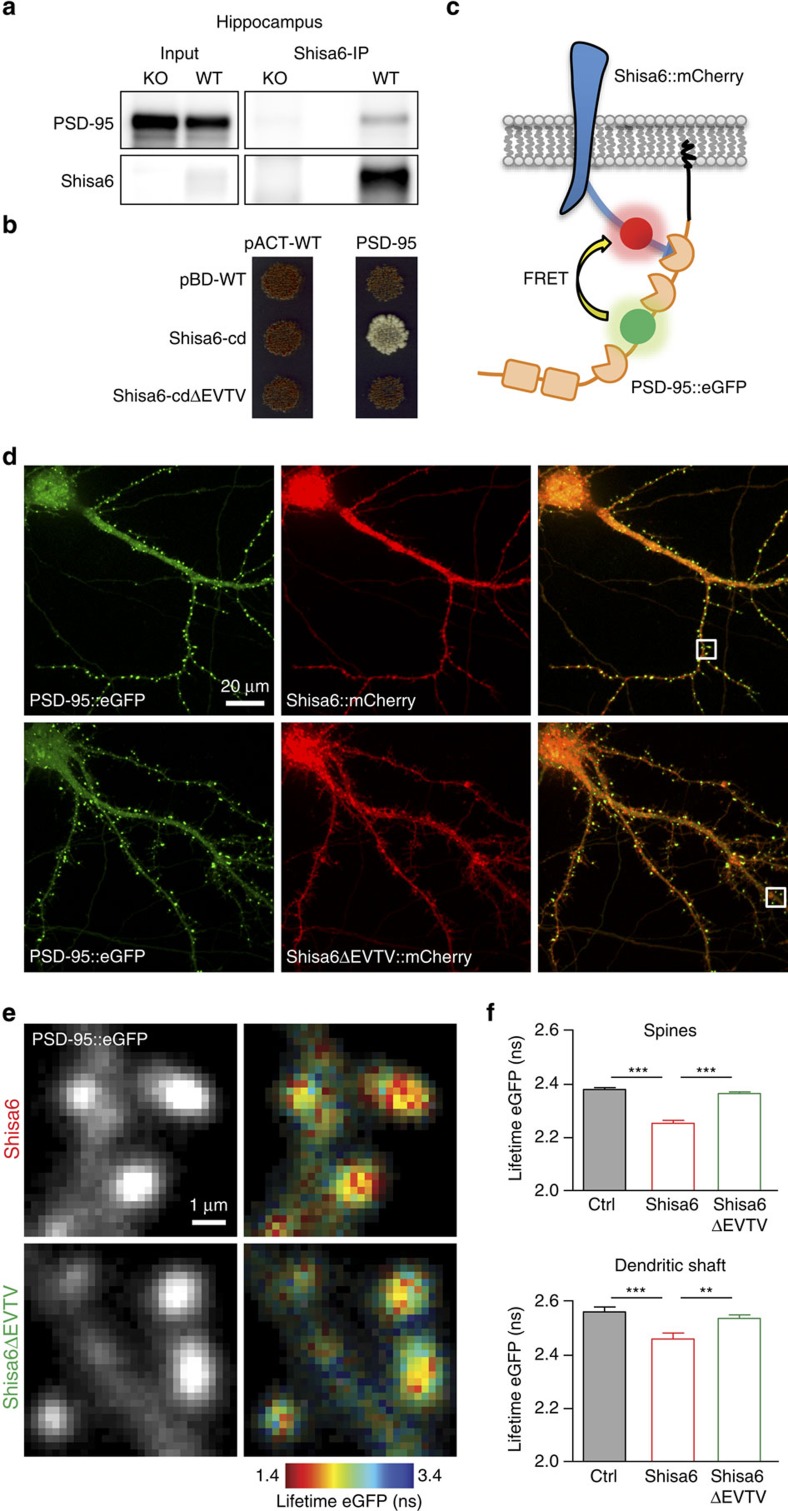
Shisa6 interacts with PSD-95 *in vitro* and in living hippocampal neurons. (**a**) PSD-95 is associated with Shisa6 in native hippocampal protein complexes on immunoprecipitation of Shisa6, and was found as the most prominent PDZ-containing interactor (Supplementary Table 1). The input controls represent 3% of the total lysate. For complete blots, in addition to those with higher exposure, see Supplementary Fig. 9. (**b**) Direct two-hybrid assay of the C-terminal part of Shisa6 (amino acids 202–557; Shisa6-cd), or with a deletion of the last four amino acids (Shisa6-cdΔEVTV), with the first two PDZ domains of PSD-95. Empty vectors (PBD-WT and pACT-WT) were used as controls. Strong cell growth was observed for the Shisa6-cd+PSD-95 condition, indicating a direct interaction. Conditions without successful bait–prey (protein–protein) interaction yielded non-growing yeast cells (red colour). (**c**) FRET design: the eGFP inserted in PSD-95 between PDZ domains 2 and 3 is in close proximity with the mCherry inserted on the intracellular C-terminal domain of Shisa6 when the two proteins are bound, and eGFP can transfer its energy to the mCherry (yellow arrow). (**d**) Sample images of neurons expressing PSD-95::eGFP (*n*=8; *N*=248 spines) and Shisa6::mCherry (*n*=10; *N*=248 spines) or Shisa6ΔEVTV::mCherry (*n*=8; *N*=442 spines). (**e**) Sample images showing dendrites with dendritic spines (left) and the same images in which each pixel is colour-coded with its corresponding eGFP lifetime value (right). (**f**) Lifetime of eGFP (mean±s.e.m.) is decreased (analysis of variance: spines, F(2,935)=72.54, *P*<0.0001; dendritic shafts, F(2,131)=7.97, *P*=0.0005) in spines (upper panel) and dendritic shafts (lower panel) of neurons expressing Shisa6::mCherry. This effect is not observed in neurons expressing Shisa6ΔEVTV::mCherry. *Post hoc* Newman–Keuls test: **P<0.010, ***P<0.001.

Given that Shisa6 co-localizes synaptically with AMPARs, we tested whether it can directly interact with the organizers of the PSD, that is, PDZ-containing proteins. First, we identified all PDZ-containing proteins within immuno-isolated hippocampal Shisa6 protein complexes, thereby identifying the scaffolding protein PSD-95 (Dlg4) as a prominent PDZ-containing interactor of Shisa6 (Fig. 3a and Supplementary Table 1). Second, using a direct yeast two-hybrid assay, we confirmed that Shisa6 is able to directly interact with PSD-95, and that binding is dependent on the C-terminal EVTV domain (Fig. 3b).

On the basis of these results, we developed a Förster resonance energy transfer (FRET) approach^25^ to assess the subcellular localization of the interaction between Shisa6 and PSD-95. A FRET pair between PSD-95::eGFP (FRET donor) and Shisa6::mCherry (FRET acceptor) was designed (Fig. 3c). Overexpression of PSD-95::eGFP and Shisa6::mCherry in cultured hippocampal neurons (Fig. 3d) and fluorescence lifetime imaging microscopy (FLIM) was then used to measure the difference of FRET through the decrease in eGFP lifetime compared with control neurons overexpressing PSD-95::eGFP only (Fig. 3e). We observed a robust FRET between PSD-95::eGFP and Shisa6 WT::mCherry in dendritic spines (lifetime eGFP in ns: control, 2.381; Shisa6, 2.254; *P*<0.001) and dendritic shaft (control, 2.563; Shisa6, 2.461; *P*<0.001) of living neurons that differed from control (*P*<0.001; Fig. 3e,f). Importantly, we observed no difference in FRET from control on expression of Shisa6ΔEVTV in both these compartments (dendritic spines, Shisa6ΔEVTV: 2.366; dendritic shaft, Shisa6ΔEVTV: 2.538; Fig. 3e,f). Thus, Shisa6 interacts with PSD-95 in dendritic spines and the dendritic shaft via a binding on the PDZ domains of PSD-95.

### Shisa6 reduces AMPAR mobility based on PDZ interaction

**Figure 4:**
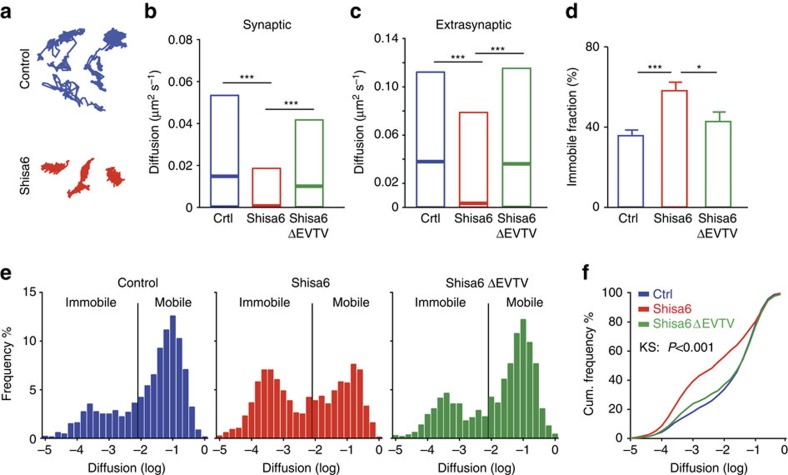
Shisa6 decreases AMPAR mobility through its PDZ-binding consensus sequence. (**a**) Representative trajectories of QD-GluA2 membrane diffusion in control hippocampal neurons (blue) or in hippocampal neurons expressing Shisa6 protein (red). (**b**,**c**) Median diffusion of GluA2 subunits in control (Crtl) hippocampal neurons (blue) or in neurons expressing Shisa6 (red) or Shisa6ΔEVTV (green). Displayed are results for the synaptic domain (**b**), labelled by Homer1c-GFP (control, *n*=311 QDs; Shisa6, *n*=133 QDs; Shisa6ΔEVTV, *n*=171 QDs) and the extrasynaptic domain (**c**, control, *n*=2126 QDs; Shisa6, *n*=865 QDs; Shisa6ΔEVTV *n*=1109 QDs), as tested by Kruskal–Wallis test. (**d**) Mean proportion of immobile QD-GluA2 in control condition (35.58%±2.84, *n*=35 neurons) or after expression of either Shisa6 (57.94%±4.35, *n*=24 neurons) or Shisa6ΔEVTV (42.95%±4.72, *n*=19 neurons). Shisa6 increases the immobile pool of receptors, whereas expression of Shisa6ΔEVTV has no effect, as tested by Bonferroni's multiple comparisons test. (**e**) Frequency distributions of the diffusion coefficient calculated from the pooled synaptic and extrasynaptic trajectories of QD-GluA2 in control or after expression of Shisa6 or Shisa6ΔEVTV. Expression of Shisa6ΔEVTV increases the diffusion coefficient to values comparable to the control conditions (**b**,**c**). (**f**) Cumulative (Cum.) distribution of the diffusion coefficient of QD-GluA2 in control neurons (blue) or in neurons expressing Shisa6 (red) or Shisa6ΔEVTV (green), with those for Shisa6 being significantly different (*P*<0.001; Kruskal–Wallis test) from those for control. All values were obtained from four independent experiments. All tests: *P<0.050, ***P<0.001.

Shisa6 is interacting with the AMPAR and binds synaptically enriched PDZ-containing scaffold proteins such as PSD-95 through its C-terminal EVTV motif. Since PSD-95 is a rather stable protein^26^, Shisa6 might stabilize AMPARs at PSD-95-enriched domains such as synapses. To assess this, we tracked in real-time the movement of native GluA2-containing AMPARs at the surface of cultured hippocampal rat neurons (DIV12), using quantum dots (QDs) coupled to specific antibodies directed against the extracellular domain of GluA2 (Fig. 4). We expressed Homer1C-GFP to label synaptic compartments either alone (control) or with Shisa6.

As previously described^27^,^28^, AMPARs exhibit different surface diffusion movements, ranging from immobile to diffusing freely, and trapped within confined domains. Representative trajectories from GluA2 showed that Shisa6 significantly decreases GluA2 mobility (Fig. 4a) in both synaptic (Fig. 4b; diffusion coefficient (μm^2^ s^−1^): control, 0.0128 (±0.0005/0.049 interquartile range (IQR)); Shisa6, 0.0006 (±0.0001/0.008 IQR); *P*<0.0001) and extrasynaptic compartments (Fig. 4c; control, 0.0378 (±0.002/0.114 IQR); Shisa6, 0.0034 (±0.0002/0.115 IQR); *P*<0.0001). The frequency distribution of GluA2 trajectory diffusion coefficients revealed that expression of Shisa6 decreased the pool of mobile receptors while increasing the immobile pool (Fig. 4d–f). After expression of Shisa6, the immobile fraction (57.94%±4.35) was higher than in control conditions (35.58%±2.85, *P*<0.001; Fig. 4d). In conclusion, expression of Shisa6 decreases GluA2 surface mobility in both the extrasynaptic and synaptic compartments.

To study the impact of interactions with PDZ-containing proteins, we performed GluA2 diffusion experiments in neurons expressing Shisa6 with the last four amino acids deleted (Shisa6ΔEVTV; Fig. 4b–f). Expression of Shisa6ΔEVTV increases the mobility of GluA2 compared with WT Shisa6 both in the synaptic (Fig. 4b; diffusion coefficient (μm^2^ s^−1^): 0.0082 (±0.0002/0.041 IQR) *P*<0.001) and extrasynaptic compartments (Fig. 4c; 0.0363 (±0.0008/0.11 IQR) *P*<0.001) bringing it to non-transfected control levels. Furthermore, the proportion of immobile receptors in the presence of Shisa6ΔEVTV was not significantly different from control cells (Fig. 4d; 42.95%±4.72; *P*=0.191) and lower than cells expressing WT Shisa6 (*P*=0.025; Fig. 4d; F(2,75)=9.69, *P*=0.0002). This effect was similarly apparent on the frequency plot of diffusion coefficient distribution (Fig. 4e). Finally, the cumulative distribution curve comparing the distributions of the two experimental and control situations (Fig. 4f) showed that expression of Shisa6 immobilizes GluA2-containing AMPARs via an interaction through its PDZ ligand.

### Shisa6 modulates AMPAR fast kinetics in HEK293 cells

**Figure 5:**
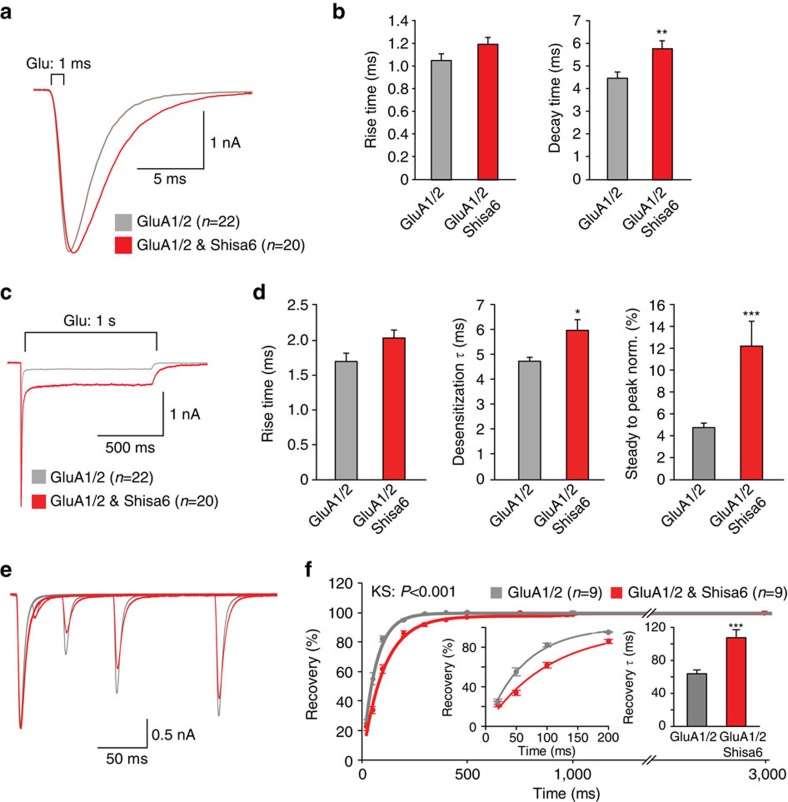
Shisa6 decreases AMPAR deactivation rate and desensitization rate, enhances the steady-state current and slows recovery from desensitization. (**a**) Peak-scaled example traces of whole-cell recording from HEK293 cells expressing heteromeric AMPAR channels in the absence (grey) or presence (red) of Shisa6. Currents were evoked by direct application of 1 mM glutamate during 1 ms. (**b**) Bar graphs (mean±s.e.m.) summarize changes in rise time (1.06±0.06 versus 1.20±0.06 ms, *P*=0.101) and decay time (4.50±0.28 versus 5.81±0.35 ms, *P*=0.005) of AMPAR currents mediated by heteromeric AMPARs in HEK293 cells in the presence and absence of Shisa6. (**c**) Peak-scaled example trace of whole-cell recordings from HEK293 cells expressing a heteromeric AMPAR channel in the absence (grey) or presence (red) of Shisa6. Currents were evoked by direct application of 1 mM glutamate during 1 s. (**d**) Bar graphs (mean±s.e.m.) summarize changes in rise time (1.60±0.08 versus 2.04±0.12 ms, *P*=0.072), desensitization time constant and steady-state AMPAR-mediated currents. (norm., normalized) *P<0.050, **P<0.010, ***P<0.001 (*t*-test). (**e**) Example trace of repeated 1-ms glutamate application from HEK293 cells expressing a heteromeric AMPAR channel in the absence (grey) or presence (red) of Shisa6. (**f**) Recovery of desensitization (two 1-ms glutamate application with inter-pulse interval of 20, 50, 100, 200, 300, 400, 500, 750, 1,000 and 3,000 ms) from HEK293 cells expressing a heteromeric AMPAR channel in the absence (grey) or presence (red) of Shisa6. Inset (left) shows recovery up to 200 ms. In the presence of Shisa6, recovery is slower, yielding an increase in *τ*_recovery_ (right inset). ***P<0.001.

Since Shisa6 and the AMPAR are partners of the same hippocampal protein complex, they interact *in vitro*, and Shisa6 traps AMPARs synaptically in neurons, we examined whether Shisa6 affects biophysical properties of AMPARs. AMPAR-mediated currents were measured in response to glutamate applications in the presence and absence of Shisa6 in HEK293 cells. Expression of Shisa6 in HEK293 cells by itself did not give rise to a glutamate-induced current on glutamate application (Supplementary Fig. 4). Co-expression of Shisa6 and AMPAR subunits prolonged the decay time of homomeric GluA1 currents, homomeric GluA2 currents, as well as GluA1–GluA2 heteromeric AMPAR currents, induced by a 1-ms glutamate application (Fig. 5a,b and Supplementary Fig. 4). AMPAR current rise times remained unchanged in the presence of Shisa6. Unlike other AMPAR modulatory proteins^29^, Shisa6 did not alter the rectification properties of heteromeric and homomeric AMPARs (Supplementary Fig. 4). In addition, Shisa6 did not alter properties of GluK2 kainate receptors (Supplementary Fig. 5).

### Shisa6 affects AMPAR slow kinetics in HEK293 cells

Since AMPAR decay time is prolonged by Shisa6, and deactivation and desensitization are closely related processes, we next investigated whether Shisa6 modulates AMPAR currents in response to prolonged desensitizing glutamate application (1 s, 1 mM) in HEK293 cells (Fig. 5c,d). In the presence of Shisa6, both heteromeric GluA1–GluA2 and homomeric GluA1-containing AMPARs displayed slower desensitization kinetics (Fig. 5d and Supplementary Fig. 4; desensitization *τ* (ms) GluA1–GluA2: 4.78±0.16 versus 6.02±0.43, *P*=0.014) and reduced desensitization, observed as an enhanced steady-state conductance in response to 1-s applications of glutamate (Fig. 5d and Supplementary Fig. 4; % of peak conductance; GluA1–GluA2, 4.59±0.04 versus 12.25±2.28, *P*<0.001). AMPAR current rise times remained unchanged for both receptor types (Fig. 5d and Supplementary Fig. 4).

We next investigated whether Shisa6 affects recovery from desensitization of heteromeric GluA1–GluA2 AMPARs using two consecutive 1-ms glutamate (1 mM) applications with variable interval (Fig. 5e,f). Shisa6 slowed down recovery from desensitization, showing an increase in the time constant of recovery (*τ*_recovery_ GluA1–GluA2, 63.78±4.64 versus 107.47±9.63 ms, *P*<0.001).

### Shisa6 alters AMPAR current kinetics in hippocampus slices

**Figure 6:**
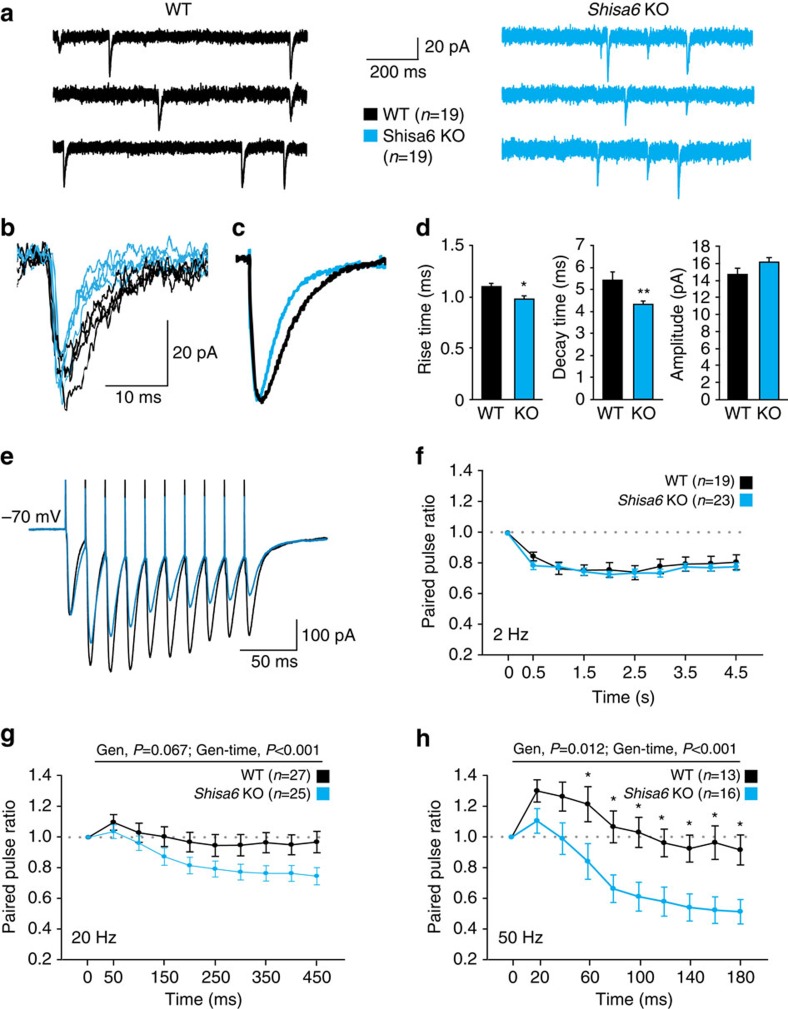
Shisa6 prolongs synaptic AMPAR currents and reduces synaptic depression. (**a**) Example traces of mEPSC recordings from CA1 pyramidal cells of *Shisa6* KO animals and WT littermates. (**b**,**c**) Superimposed spontaneous synaptic currents (**b**), and average synaptic currents (**c**) of *Shisa6* KO animals and WT littermates. (**d**) Bar graphs (mean±s.e.m.) of *Shisa6* KO and WT animals (*n*=19 cells per genotype, from four WT and four KO animals) represent rise time and decay time of mEPSCs, showing that both parameters are affected *ex vivo*. Amplitude was not significantly affected (16.21±0.52 versus 14.73±0.65 pA, *P*=0.085). (**e**) Superimposed example traces of whole-cell recording voltage clamped at −70 mV from CA1 pyramidal neurons of *Shisa6* KO (blue) animals and WT littermates (black) in response to 50-Hz stimulation of synaptic inputs from Schaffer collateral fibres. (**f**–**h**) Pulse ratios of electrically evoked EPSCs from CA1 pyramidal neurons (at −70 mV) of *Shisa6* KO (from five animals) animals and WT littermates (from six animals) at 2 (**f**), 20 (**g**) and 50 (**h**) Hz. At 20 Hz there was a trend for genotype effect (Gen, F(1,450)=3.52, *P*=0.067), and a significant effect of time (F(9,450)=17.81, *P*<0.001), as well as a genotype × time interaction (Gen-time, F(9,450)=3.91, *P*<0.001). The 50-Hz trains revealed a significant genotype effect (F(1,243)=7.33, *P*=0.012), time effect (F(9,243)=39.87, *P*<0.001) and a genotype × time interaction (F(9,243)=6.29, *P*<0.0001). Cell numbers used are indicated. *P<0.050 (Bonferroni *post hoc* test).

To test whether Shisa6 affects AMPAR function in the hippocampus, we recorded AMPAR spontaneous miniature excitatory postsynaptic currents (mEPSCs) in CA1 pyramidal cells in acute hippocampal slices of *Shisa6* WT and KO mice (Fig. 6a–d). In WT pyramidal neurons, both the rise and decay kinetics of mEPSCs were slower than in KO neurons (rise time (ms): 1.10±0.03 versus 0.98±0.03, *P*=0.013; decay time (ms): 5.43±0.36 versus 4.27±0.15, *P*=0.007). There was no significant difference in mEPSC amplitude and frequency (Fig. 6d and Supplementary Fig. 6). Immunoblotting of the hippocampal synaptic membrane fraction from *Shisa6* WT and KO mice revealed no difference in the number of (subunits of) the AMPAR, NMDAR, PSD-95, TARPs or CKAMP44/Shisa9 present at the synapse (Supplementary Fig. 6). These findings show that the presence of Shisa6 specifically alters the kinetics of AMPAR synaptic currents.

We next investigated whether the Shisa6 effects on AMPARs play a role in short-term synaptic plasticity and prolonged exposure to glutamate in the hippocampus in CA1 pyramidal cell dendrites. To that end, we first tested the effect of prolonged glutamate application by local glutamate uncaging on hippocampal (CA1) dendrites in *Shisa6* WT and KO mice. CA1 pyramidal cell dendrites were visualized by adding Alexa-488 to the patch solution. A small, localized puff of Rubi-glutamate was applied to dendrites 1 s before uncaging with light. Light-induced currents were completely abolished by DNQX (10 μM). In the local glutamate uncaging experiments (Supplementary Fig. 7), light-induced AMPAR currents were large (100–500 pA) and had rise times that were about five times slower than synaptic currents (cf. Fig. 5a,b). Light-induced AMPAR currents typically lasted hundreds of milliseconds, with a decay time constant of about 150 ms (Supplementary Fig. 7), most likely reflecting the time course of glutamate clearing. In *Shisa6* KO animals, decay times of light-induced AMPAR currents were reduced to half of the decay times in WT animals (Supplementary Fig. 7), whereas rise times did not change. Given the slow time course of the light-induced AMPAR currents, the difference in decay time most likely results from reduced AMPAR desensitization by Shisa6 in WT animals. Interestingly, AMPAR-mediated currents elicited by rapid glutamate application to nucleated patches of CA1 pyramidal cells did not differ between WT and *Shisa6* KO conditions (Supplementary Fig. 7). This suggests that Shisa6 does not functionally regulate somatic AMPARs, in agreement with our observation that cell bodies do not stain for Shisa6 (Figs 1 and 2).

Second, we tested whether Shisa6-induced modifications of AMPAR function affect frequency-dependent short-term synaptic plasticity. We stimulated Schaffer collaterals at different frequencies during whole-cell recordings from CA1 pyramidal neurons to repeatedly activate glutamatergic inputs to these neurons, while blocking GABARs with gabazine (Fig. 6e). With only two stimulation pulses, we did not observe significant differences in the paired-pulse ratios at any stimulation frequency (Fig. 6f–h). With stimulation trains of 10 pulses at low frequencies (2 Hz; Fig. 6f), we also did not observe a change in synaptic depression. However, at 20 and 50 Hz stimulation, *Shisa6* KO synapses displayed stronger depression than WT synapses (Fig. 6g,h). To exclude the possibility of an underlying presynaptic mechanism, we tested depression of synaptic NMDAR currents with the same stimulation protocol, while inhibiting AMPAR currents with NBQX (Supplementary Fig. 8). We did not find differences in NMDAR-mediated current kinetics, neither in responses between WT and *Shisa6* KO synapses at any of the stimulation frequencies. This suggests that in WT glutamatergic synapses, AMPAR currents maintain larger amplitudes during repeated synaptic activation. Enhanced synaptic depression observed in *Shisa6* KO synapses most likely resulted from enhanced levels of AMPAR desensitization. These findings identify a role of Shisa6 in maintaining glutamatergic synaptic transmission during repeated synaptic activity.

## Discussion

We identified Shisa6 as an intrinsic auxiliary subunit of AMPAR complexes in the mammalian brain with unique characteristics compared with the CKAMP44/Shisa9 member of this family (Supplementary Table 3). Physical association of Shisa6 with the pore-forming GluA proteins modulates receptor properties by slowing synaptic AMPAR current activation and desensitization. Shisa6 traps AMPARs at the postsynapse *in vivo*, slows desensitization kinetics and favours a sustained open state on prolonged activation. Together, these processes reduce short-term synaptic depression.

Shisa6 qualifies as a *bona fide* auxiliary subunit of the AMPAR according to criteria as outlined by Yan *et al*.^30^. First, Shisa6 is a non-pore-forming subunit; expression of Shisa6 alone in HEK293 cells did not lead to a current when activated with glutamate. Second, Shisa6 has a direct and stable interaction with GluA pore-forming subunits; immunoprecipitation experiments using an anti-GluA2 antibody detected Shisa6, and reverse, anti-Shisa6 antibody confirmed the interaction between AMPARs and Shisa6, both *in vitro* and in the brain. Third, Shisa6 modulates channel properties: both *in vitro* experiments and gene deletion of Shisa6 *in vivo* led to affected kinetics and desensitization properties of AMPAR currents. In addition, Shisa6 affected AMPAR mobility. Fourth, Shisa6 is necessary *in vivo*: gene deletion of Shisa6 showed affected rise and decay times of AMPAR currents in the hippocampus, and affected AMPAR-dependent short-term synaptic plasticity.

Shisa6 limits AMPAR diffusion and induces strong AMPAR stabilization at synaptic sites. Under basal conditions, most AMPARs are not stable at synapses but alternate constantly between immobile and mobile states, and mobile AMPAR exchange between synaptic and extrasynaptic sites within seconds^27^,^28^,^31^. On average, about 50% of synaptic AMPARs are immobile at any given point in time, being concentrated in nanoscale clusters, while they are highly mobile in between these clusters^32^. AMPAR surface diffusion and synaptic stabilization are highly regulated by neuronal activity^33^,^34^ and thought to be one of the main mechanisms for activity-dependent regulation of AMPAR concentration at synapses, a process at the origin of many forms of synaptic plasticity^35^. The precise molecular mechanisms of the activity-dependent, reversible AMPAR stabilization at synapses are still unclear as it does not seem to directly depend on AMPAR subunits^28^. Rather, AMPAR stabilization at PSDs involves interactions of auxiliary subunits with intracellular scaffold proteins. The best-established example of the activity-dependent stabilization of AMPARs is through binding of the C-terminus of the auxiliary subunit TARP gamma2 (also called Stargazin) to PSD-95, mediated by CaMKII-dependent Stargazin phosphorylation^15^,^33^,^36^. At rest, reversible binding between Stargazin and PSD-95 allows AMPARs to alternate between diffusive and immobile states, and synaptic trapping of pre-existing surface receptors through rapid CaMKII-induced phosphorylation of TARPs is proposed to be one of the first events during synaptic potentiation^15^,^33^,^34^,^35^. Here we show by using both single-molecule tracking of AMPAR movement and FRET between Shisa6 and PSD-95 that Shisa6 can also bind to PSD-95 and immobilize AMPARs. Interestingly, although Shisa6 is accumulated at synaptic sites, it can immobilize AMPARs both at synaptic and extrasynaptic sites, most likely through binding to synaptic and extrasynaptic scaffolds.

Whereas Stargazin is present at saturated levels in the synapse under basal conditions^28^, only a portion of AMPAR interaction sites is occupied by native Shisa6, because Shisa6 overexpression still has the capacity to decrease mobility. The presence of additional Shisa6–AMPAR interaction sites in the synapse is substantiated by the absence of a dominant negative effect of Shisa6–ΔEVTV. It is unlikely that Shisa6 competes with Stargazin/TARP for AMPAR binding, and by doing so would be more effective in reducing AMPAR mobility than Stargazin/TARP, as one would expect that overexpression of the non-immobilizing Shisa6–ΔEVTV protein would also lead to replacement of WT Stargazin/TARP. This condition would mimic that created on expression of non-functional stargazin leading to a dominant negative effect^28^. As we did not observe an increase in mobility compared with the basal/control condition, we conclude that Shisa6 is likely to bind the AMPAR complementary to stargazin/TARP, as has been observed for Shisa9 (ref. 37). The fact that we find TARP in the immunoprecipitated complex of Shisa6 is in agreement with this. Whether Shisa6–AMPAR binding to PSD-95 is distinctly regulated by neuronal activity from TARP–AMPAR binding to PSD-95 will be interesting to determine.

Shisa6 interacts with AMPAR complexes in the hippocampus that contain TARPγ-8, but that do not contain CKAMP44/Shisa9. Interestingly, Khodosevich *et al*.^37^ reported that CKAMP44/Shisa9 and TARPγ-8 coexist on the same AMPAR complexes in the dentate gyrus. CKAMP44/Shisa9 is thus not likely to decorate the same AMPAR population as Shisa6. These findings might further underline the differential cellular function of both proteins even when both are present in the dentate gyrus. Shisa6 slows entry of AMPARs into the desensitized state and increases steady-state currents in the prolonged presence of glutamate. This may be viewed as stabilization of the open state by impairing channel closure probably induced by a conformational process. In that respect, Shisa6 is analogous to TARPs and CNIH in its action on AMPARs. This is in contrast with the effect of its homologue CKAMP44/Shisa9 that facilitates entry into the desensitized state and decreases the steady-state current^18^. Noteworthy, Shisa6 reduces the rate of recovery from desensitization, similarly to, but to a lesser extent than CKAMP44/Shisa9. The slower rate of recovery from desensitization induced by Shisa6 that we observed in HEK293 cells seems at odds with the increased synaptic depression we observed in the KO. If recovery from desensitization is a dominant factor in synaptic depression, then we would have expected Shisa6 to increase synaptic depression. However, it did not. In WT animals we observed much less synaptic depression. It is therefore likely that the reduced rate of desensitization and an increased sustained AMPAR current induced by Shisa6, as we observed in HEK293 cells, underlies the reduced synaptic depression in WT synapses. Whereas deletion of Shisa6 modifies mEPSC kinetics, which is in agreement with Shisa6 overexpression in HEK293 cells, deletion of *CKAMP44/Shisa9* alters AMPAR mEPSC amplitude and frequency with no effect on mEPSC kinetics.

The most striking difference between Shisa6 and CKAMP44/Shisa9 is that in response to trains of synaptic activation, CKAMP44/Shisa9 reduces synaptic facilitation, whereas we find that Shisa6 reduces synaptic depression. This indicates that the Shisa6-induced slowing down of AMPAR entry in the desensitized state overcomes the Shisa6-induced slowing down of recovery from desensitization in controlling short-term synaptic plasticity.

Although both Shisa6 and CKAMP44/Shisa9 seem to interact with proteins of the postsynaptic density, the exact physiological relevance of this interaction is not yet understood. We found that in the presence of Shisa6, AMPARs are restricted in their synaptic movement, without changing the number of AMPARs on the synaptic surface. Since CKAMP44/Shisa9 was reported to increase the amplitudes of evoked AMPAR currents and to promote surface expression in overexpressing cells^37^, these findings indicate a role in the surface trafficking of CKAMP44/Shisa9-decorated AMPARs, with as yet unknown effect on membrane mobility of AMPARs.

We showed previously that AMPAR surface mobility is key to recovery from frequency-dependent synaptic depression at glutamatergic synapses by allowing the exchange of desensitized AMPARs for naive ones^38^. Activity-dependent immobilization of AMPARs at synaptic sites leads to increased desensitization of glutamatergic synaptic currents during paired-pulse stimulation, resulting in stronger synaptic depression^33^,^38^. Synapses thus have to face the conundrum that by having more stable AMPARs, for example, after potentiation, they become sensitive to high-frequency-induced depression due to AMPAR cumulative desensitization. We found that synaptic AMPARs trapped by Shisa6 are less desensitized by repeated synaptic activation. This sustained activated state in the presence of glutamate might serve as a Shisa6-mediated mechanism to protect synaptic AMPARs from full desensitization on repeated synaptic activity. Expression of Shisa6 thus allows synapses to sustain higher transmission rates by preventing AMPAR desensitization.

## Methods

### Animals

Mice were bred in the facility of the VU University Amsterdam. Mice were group-housed in standard type 2 Macrolon cages enriched with nesting material on a 12/12-h rhythm (lights on at 07:00). The housing area had a constant temperature of 23±1 °C and a relative humidity of 50±10%. Food and water were provided *ad libitum*. All the experiments were performed between 09:00 and 17:00. Protein samples and RNA were prepared from 8- to 14-week-old male and female C57/BL6J mice, derived from Charles River. Immunoprecipitations were performed on hippocampi of 8- to 14-week-old male and female WT versus KO mice. Electrophysiology on CA1 neurons was performed on 8- to 12-week-old males. All experiments were performed in accordance to Dutch law and licensing agreements using a protocol approved by the Animal Ethics Committee of the VU University Amsterdam. All our protocols have been performed in accordance with the recommendations of the European directive EU-2010-63 for the raising, care and termination of animals. In Bordeaux the maintenance of animals was supervised by the Pole *in vivo* facility. For generation of *Shisa6* KO mice, see Supplementary Methods.

### (Real-time) PCR

*Primers.* Primers for PCR and real-time PCR were generated using Primer3.0. The final sets of primers are listed in Supplementary Table 2.

*RNA isolation and cDNA synthesis*. RNA from several tissues was extracted as previously described^39^ (supplementary Methods).

*PCR for exon 3.* PCR reactions on two WT samples were generated with Ex1–Ex6 primers (Supplementary Table 2) with 0.5 U Phusion (New England Biolabs) in a 50-μl reaction using the HF buffer according to the manufacture's protocol (Supplementary Methods).

*Real-time quantitative PCR*. Real-time quantitative PCR reactions were performed as previously described^39^ (Supplementary Methods).

### Immunoblot analysis

Protein samples were dissolved in SDS sample buffer (Laemmli), heated to 96 °C for 5 min, and loaded onto a 4–15% Criterion TGX Stain-Free Precast gel (Bio-Rad). The gel-separated proteins were imaged with the Gel-Doc EZ system (Bio-Rad), directly transferred onto polyvinylidene difluoride membrane and probed with various antibodies (Supplementary Methods). Scans were acquired with the Odyssey Fc system (Li-Cor), and analysed using Image Studio 2.0 software (Li-Cor). Immunoblot band intensities were normalized to the total amount of protein loaded, as quantified using Image Lab 3.0 software (Bio-Rad).

### Subcellular fractionation

Subcellular fractions were prepared as described previously^19^, with some modifications (Supplementary Methods).

### Precipitation of protein complexes from mouse hippocampus

Precipitation of protein complexes from mouse hippocampus was carried out as described in Supplementary Methods.

### Co-precipitation from HEK293 cells

All steps were performed at 4 °C, with the exception of elution (room temperature). For protein extraction, HEK293 cells (ATCC) were washed with PBS, resuspended in lysis buffer (1% Triton-X100, 150 mM NaCl, 25 mM HEPES and EDTA-free complete protease inhibitor (Roche), and incubated for 1 h with gentle mixing. The supernatant was cleared of non-soluble debris by two consecutive centrifugation steps at 20,000*g* for 20 min. Anti-flag antibody was added to the supernatant, incubated O/N, and immobilized to Protein A/G agarose beads (Santa Cruz). The agarose beads were washed four times with lysis buffer, and bound proteins were eluted by incubation with Laemmli sample buffer.

### Yeast two-hybrid

A direct two-hybrid assay was performed in PJ69-2A yeast cells (Clontech) between the WT cytoplasmic domain of the exon 3 containing form of Shisa6 (Shisa6-cd WT, amino acids 202–557) or the truncated mutant thereof (Shisa6-cd ΔEVTV; both contained within the pBD-Gal4 vector (Stratagene)), and PSD-95 (amino acids 39–262 of NP_031890.1, encoding PDZ domains 1 and 2; contained within the pACT2 vector (Clontech)) as described^19^. Empty pBD-Gal4-WT and pACT2-WT vectors were used as matching controls. Cell growth was recorded after 4 days of stringent nutritional selection (−Leu, −Trp, −His and −Ade). Methods as described in ref. 19.

### Dissociated hippocampal neuronal cultures

Hippocampal neurons derived from 18-day-old rat embryos of either sex were cultured following the Banker protocol^40^. Briefly, dissociated neurons were plated on poly-L-lysine-coated glass coverslips at a density of ∼18,000 cells per cm^2^ and co-cultured over an astroglial feeder layer in Neurobasal medium supplemented with B27.

### Immunocyto- and histochemistry

For culture staining, anti-GluA2 and anti-flag antibodies were applied on live neurons (DIV16), which were then fixed in 4% paraformaldehyde and permeabilized with 0.2% Triton-X100 before incubation with anti-PSD-95. Then, cells were rinsed and incubated with appropriate Alexa-conjugated secondary antibodies (Invitrogen; see Supplementary Methods for details). Images of triple-stained neurons were obtained by epifluorescence microscopy (Leica, DM5000). For slice staining, we used the method of Yoneyama *et al*.^41^.

### Single-nanoparticle tracking of surface AMPARs

The experimenter was blind to the construct used, which was revealed after analysis. For endogenous GluA2 QD tracking, rat hippocampal neurons were incubated with monoclonal antibody directed against N-terminal extracellular domain of GluA2 subunit for 10 min followed by 5-min incubation with QDs 655 Goat F(ab')2 anti-mouse IgG (Invitrogen). QDs were detected by using a mercury lamp and appropriate excitation/emission filters. Images were obtained with an interval of 50 ms and up to 1,000 consecutive frames. Signals were detected using a CCD (charge-coupled device) camera (Quantem; Roper Scientific). QD recording sessions were processed with the Metamorph software (Universal Imaging Corp.). The instantaneous diffusion coefficient *D* was calculated for each trajectory, from linear fits of the first four points of the mean square displacement versus time function using MSD(*t*)=<*r*^2^> (*t*)=4*Dt*. The two-dimensional trajectories of single molecules in the plane of focus were constructed by correlation analysis between consecutive images using a Vogel algorithm. QD-based trajectories were considered synaptic if co-localized with Homer 1C dendritic clusters for at least five frames.

### Fluorescence lifetime imaging microscopy experiments

The experimenter was blind to the construct used, which was revealed after analysis. FLIM experiments were performed at 37 °C using an incubator box with an air heater system (Life Imaging Services) installed on an inverted Leica DMI6000B (Leica Microsystem) spinning disk microscope and using the LIFA frequency domain lifetime attachment (Lambert Instruments) and LI-FLIM software. Cells were imaged with a HCX PL Apo × 100 oil numerical aperture (NA) 1.4 objective using an appropriate green fluorescent (GFP) filter set. Cells were excited using a sinusoidally modulated 1-W 477-nm LED (light-emitting diode) at 40 MHz under wild-field illumination. Emission was collected using an intensified CCD LI2CAM camera (FAICM, Lambert Instruments). The phase and modulation were determined from a set of 12 phase settings using the manufacturer's software LI-FLIM software. Lifetimes were referenced to a 1-mM solution of fluorescein in saline (pH 10) that was set at 4.00 ns lifetime.

Following FLIM measurements, cells were excited using a 100-mW 491-nm DPSS laser (Calypso, Cobolt) for GFP imaging and a 100-mW 561-nm DPSS lasers (Jive, Cobolt) for imaging, and imaged with a HCX PL Apo CS × 63 oil NA 1.4 objective. Signals were recorded with a back-illuminated Evolve EMCCD camera (Photometrics). Acquisitions were done with the software MetaMorph (Molecular Devices).

### HEK293 cell transfection and coverslipping

HEK293 cells were transfected with plasmid DNA (0.8 μg) using polyethylenimine (25 kDa linear, Polysciences) and incubated for 24–48 h after transfection. Cells were passaged at least 3 h before transfection in DMEM media (Gibco), 10% FBS (Invitrogen), 1% penicillin–streptomycin (Gibco, Life Technologies) in 2.5-cm dishes. Cells were 60–70% confluent when transfected. Three hours before recording, cells were transferred to coverslips that were coated with 100 μg ml^−1^ poly-L-lysine (Sigma Aldrich). To ensure consistency in the culture, we used HEK293 cells that were passaged no more than 24 times.

### Electrophysiological recordings from HEK293 cells

For electrophysiological recordings from HEK293 cells, refer to Supplementary Data.

### Electrophysiology in acute hippocampal brain slices

Mice were decapitated and the brain was removed from the skull in ice-cold slice solution containing (in mM): 126 NaCl, 3 KCl, 10 D-glucose, 26 NaHCO_3_, 1.2 NaH_2_PO_4_, 0.5 CaCl_2_ and 7 MgSO_4_. Acute horizontal hippocampal slices were cut with a thickness of 300 μm, using a vibratome (Microm HM 650 V) in ice-cold slice solution and transferred to standard artificial cerebro-spinal fluid (aCSF) for a recovery period of at least 1 h before recordings.

Glass electrodes of 3–5 MΩ resistance were used for all whole-cell recordings from acute brain slices and pulled using borosilicate glass (outer diameter 1.5 mm, inner diameter 0.86 mm; Harvard Apparatus). Slice recordings were performed using standard aCSF (see above) at 32^ ^°C. During all experiments, the experimenter was blind to the genotype of the animal. Input/access resistances were monitored throughout the recordings. Unless indicated otherwise, used salts were purchased from Sigma Aldrich and drugs were purchased from Abcam. The experimenter was blind to the genotype used, which was revealed after analysis. Input resistance changes were monitored throughout the recordings. Our average input resistance for CA1 pyramidal cells was around 165 MΩ. Cells with input resistance changes above 20% within a recording were discarded before analysis. Our typical access resistance was around 10 MΩ. Cells with access resistances above 20 MΩ were not used for analysis.

*Miniature EPSCs*. CA1 pyramidal cells were patched using electrodes of 3–5 MΩ resistance, filled with intracellular solution containing (in mM): 125 Cs-Gluconate, 5 CsCl_2_, 4 NaCl, 10 HEPES, 0.2 EGTA, 2 K-Phosphocreatine, 2 Mg-ATP, 0.3 GTP and 4% Biocytin (Molekula GmbH; pH 7.3, 290 Osm). Slices were superfused with standard aCSF that was supplemented with 1 μM tetrodotoxin and 10 μM SR-95531 (Gabazine).

*Short-term plasticity*. Whole-cell recordings of CA1 pyramidal cells were achieved using intracellular solution containing (in mM): 125 Cs-Gluconate, 5 CsCl_2_, 4 NaCl, 10 HEPES, 0.2 EGTA, 2K-Phoshocreatine, 2 Mg_2_-ATP, 0.3 GTP and 4% Biocytin (pH 7.3, 290 Osm). CA1 pyramidal cells were stimulated in the stratum radiatum of the hippocampus around 80–150 μm distance to the soma at dendrite proximal locations. Dendrites were visualized using Alexa-488 (Life Technologies) in the intracellular recording solution. Electrical stimulation was performed using an extracellular stimulation electrode of 2–3 MΩ resistance, filled with standard aCSF. Moderate stimulation intensity was assessed during an I/O protocol at the beginning of each recording. Synaptic changes were recorded in response to 10 pulse presynaptic stimulation at frequencies between 2 and 50 Hz at a holding potential of −70 mV. Slices were superfused with standard 32-°C aCSF, which was supplemented with 10 μM SR-95531 (Gabazine). NMDA currents were measured as indicated above in the additional presence of 2 μM NBQX voltage clamped at −30 mV. NMDA receptors were blocked by the addition of 20 μM APV to visualize the nature of the remaining current.

### Statistics

Data are presented as mean±s.e.m. or medians±IQR defined as the interval between 25 and 75% percentile. All tests were two-sided. Replicates were biological in nature.

*Cell imaging*. Normally distributed data sets were compared using the paired Student's test and unpaired Student's *t*-test. Statistical significance (Prism 4.0 (GraphPad)) between more than two normally distributed data sets was tested by one-way analysis of variance test followed by a Newman–Keuls test to compare individual pairs of data. Non-Gaussian data sets were tested by non-parametric Mann–Whitney test.

*Electrophysiology*. Data were analysed using custom-made Matlab (Mathworks) software and MiniAnalysis (Synaptosoft) for the analysis of mEPSCs. Rise times were measured as the time that it took to get from 20 to 80% of the maximal amplitude. Current decays are reported as decay time (90–10%) when stated, otherwise the desensitization time constant that was determined by fitting double exponential curves, is reported. Statistical significance was assessed using Graphpad Prism 5 software (GraphPad Software). A Student's *t*-test was applied when data passed the Kolmogorov–Smirnov test for normality. If not, significance was determined using a Mann–Whitney *U*-test. Significance of short-term-plasticity data was assessed using a two-way analysis of variance with Bonferroni *post hoc* testing.

## Additional information

**How to cite this article**: Klaassen, R. V. *et al*. Shisa6 traps AMPA receptors at postsynaptic sites and prevents their desensitization during synaptic activity. *Nat. Commun.* 7:10682 doi: 10.1038/ncomms10682 (2016).

## Supplementary Material

Supplementary Information Supplementary Figures 1-9, Supplementary Tables 1-3, Supplementary Methods and Supplementary References.
