# Supplementary material for: Shisa6 traps AMPA receptors at postsynaptic sites and prevents their desensitization during synaptic activity
Source: Nat Commun. 2016 Mar 2;7:10682. doi: 10.1038/ncomms10682 (PMC4778035; doi:10.1038/ncomms10682)
Supplement: Supplementary Information — Supplementary Figures 1-9, Supplementary Tables 1-3, Supplementary Methods and Supplementary References. [file ncomms10682-s1.pdf]

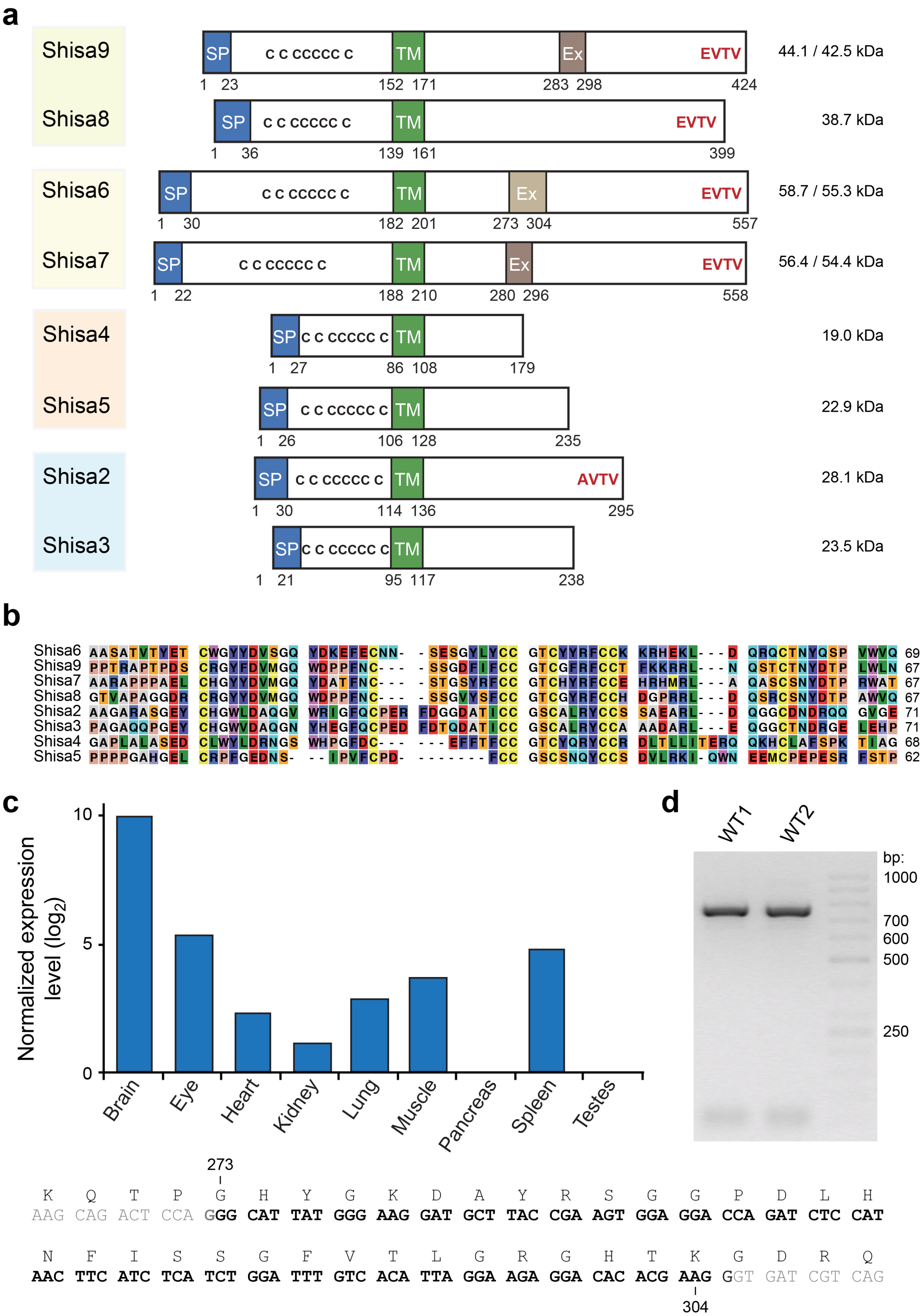

Supplementary Figure 1. Shisa6 structure and tissue expression. **a**) Schematic of the Shisa family, featuring a signal peptide (SP; blue), an extracellular domain with conserved cysteine-rich

motif, a single transmembrane region (TM; green), and an intracellular domain with a type-II[1] PDZ-ligand motif (EVTV/AVTV; red). An alternative spliced exon (Ex; brown) is present in CKAMP44/Shisa9, Shisa6 and Shisa7, albeit of different size and exon number. Shisa6 and Shisa7 form a subfamily with CKAMP44/Shisa9 and Shisa8. Shisa4 and Shisa5, as well as Shisa2 and Shisa3 form separate families[2]. Numbers under the protein indicate the beginning and end of the SP, TM and alternatively spliced exon domain, and the total amino acid length of the protein. The mature predicted molecular weight of each protein (including that of the alternative spliced variants) is indicated. **b)** Sequence alignment of the Cysteine-rich domain found in the N-terminal extracellular part of Shisa proteins, containing 8 highly conserved cysteine residues. This domain shares no proven similarity to more classical cystine-knot motifs with 6 cysteine residues found in growth factors[3,4], toxins[5] and cyclotides[6]. **c**) Quantitative PCR shows that the *Shisa6* gene is specifically enriched within the brain (note the $\log_2$-scale), and virtually absent in pancreas and testes. **d**) RT-PCR on cDNA generated from hippocampal RNA using primers flanking exon 3 of *Shisa6*. Using this RT-PCR on WT mice in duplicate, we observed a single prominent *Shisa6* transcript that contains exon 3. Sequence analysis of this PCR product confirmed the presence of exon 3 (black letters) between exons 2 and 4 (gray letters). The amino acid sequence is indicated above the nucleotide sequence. Importantly, the tryptic peptide SGGPDLHNFISSGFVTLGR, derived from exon 3 of *Shisa6*, was identified using mass spectrometric analysis of native hippocampal Shisa6 complexes (Supplementary Table 1), in three independent immunoprecipitation experiments. The exon 3-less form of *Shisa6* (GenBank NM_001034874.3/NP_001030046.1) that has been uploaded[7,8] was not detected in the hippocampus.

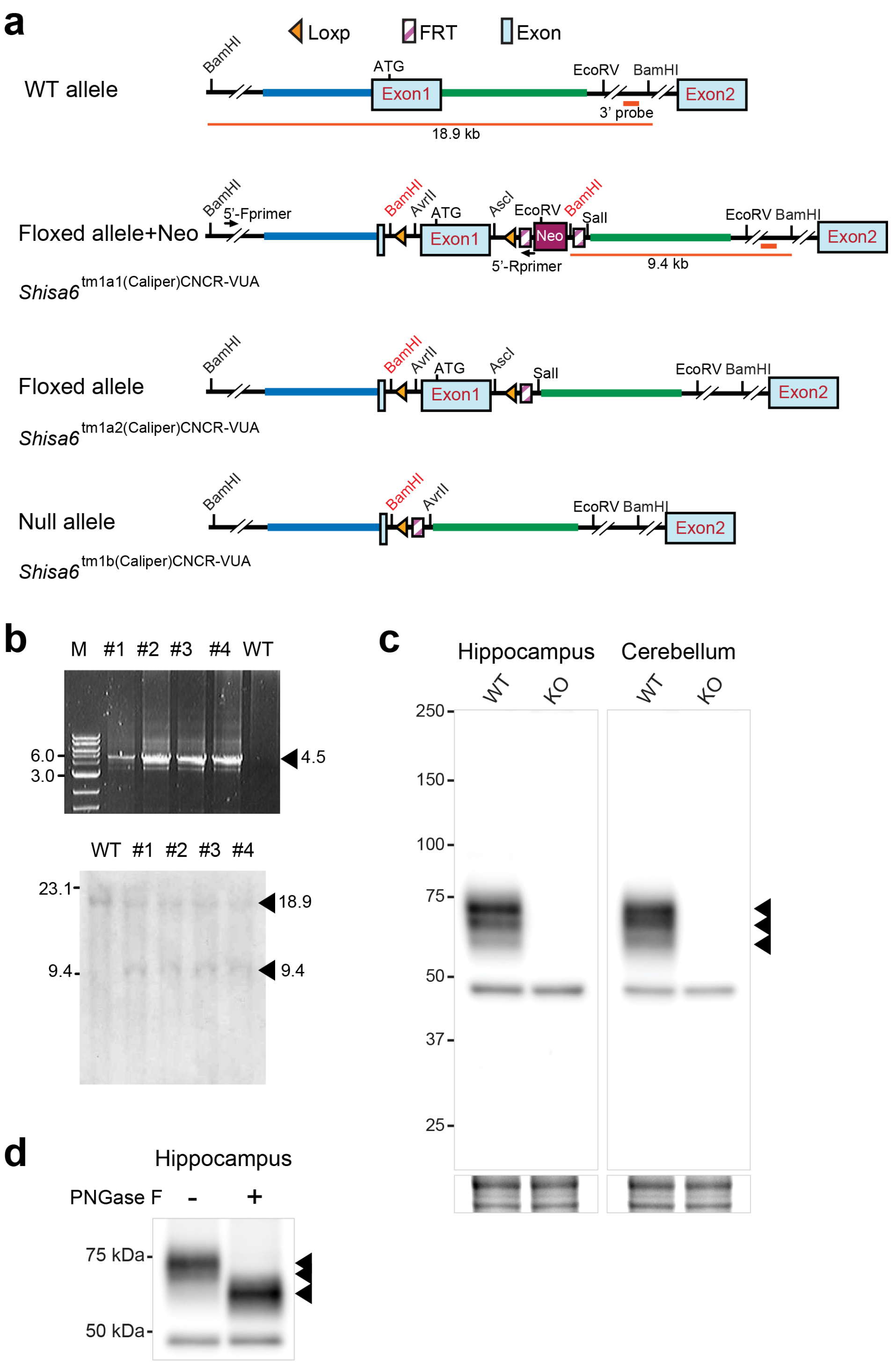

Supplementary Figure 2. Generation of *Shisa6* KO mice and antibody testing. **a**) Representation of *Shisa6-null* mouse generation: The *Shisa6* locus around exon 1, encoding the N-terminal part of the Shisa6 protein including the start-site (ATG), with essential restriction sites (red), the 5'-end PCR primer combination and the 3'-probe (and size of fragment) used for Southern blotting, is

shown (top) for the *Shisa6*$^{tm1a1(Caliper)CNCR-VUA}$ mouse line, containing the NEO cassette, the LoxP and FRT sites. The mouse line with the Neo cassette deleted (*Shisa6*$^{tm1a2(Caliper)CNCR-VUA}$) and the line with the null-allele (*Shisa6*$^{tm1b(Caliper)CNCR-VUA}$) are indicated. **b**) Correct homologous recombination in ES clones (*Shisa6*$^{tm1a1(Caliper)CNCR-VUA}$) was checked by PCR (5'-end), generating a 4.5-kB product only in mutant ES clones (indicated by the triangle; *left panel*), which was confirmed by sequencing, and by Southern blot (3'-end) after BamHI digestion, generating a 18.9-kB fragment in WT and mutants, and a 9.4-kB fragment in mutants (indicated by triangles; *right panel*). **c**) Specificity of our in-house raised Shisa6 antibody for immunoblotting was confirmed by absence of signal in hippocampal and cerebellar crude synaptic membrane fractions from *Shisa6* KO animals. Arrowheads indicate the apparent molecular weights of endogenous Shisa6. **d**) The observed molecular weight of hippocampal Shisa6 under reduced and denatured (SDS-PAGE) conditions (~73 kDa) was higher than expected based on theoretical protein size alone (predicted mature molecular weight of 58.7 kDa), potentially due to protein glycosylation. Treatment with PNGase-F reduced the observed molecular weight substantially to a single band of ~59 kDa, confirming the presence of N-linked glycans on native Shisa6 (for complete blots, see Supplementary Fig. 9).

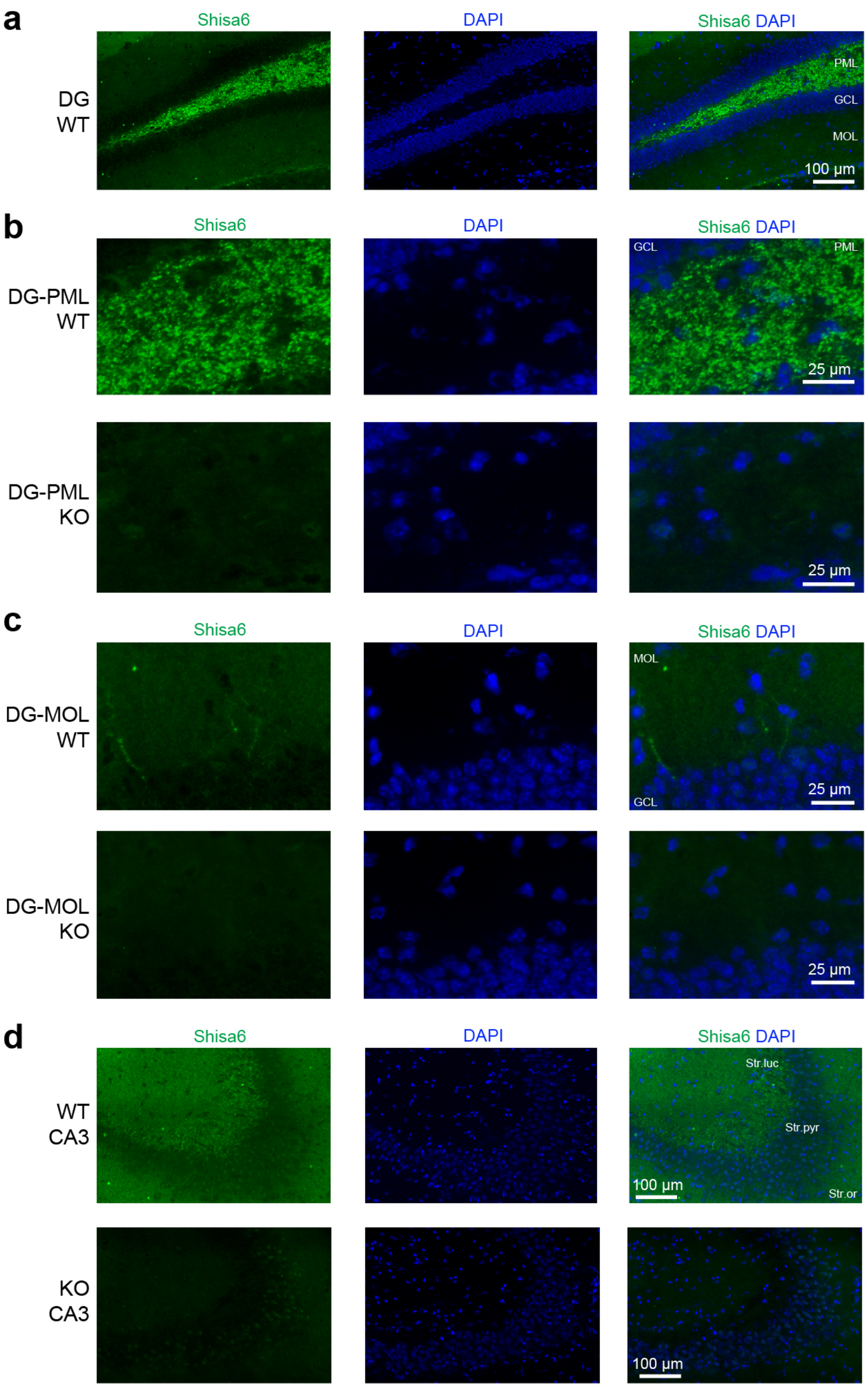

Supplementary Figure 3. Shisa6 shows specific staining in dendrites of the hippocampus. Immunostaining in WT brains for Shisa6 (green) and DAPI (blue) in the DG (a–c), and the CA3

(d) regions. **a**) An overview of the DG is shown. **b,c**), Zoom-in of the polymorphic layer (b) and the molecular layer (c) with comparison of immunostaining in *Shisa6* KO brains. **d**) Zoom-in of the CA3 area. Scale bars, and cell layers are indicated (PML, polymorphic layer; GCL, granular cell layer; MOL, molecular layer; Str.luc, stratum lucidum; Str.pyr, stratum pyramidale; Str.or, stratum oriens).

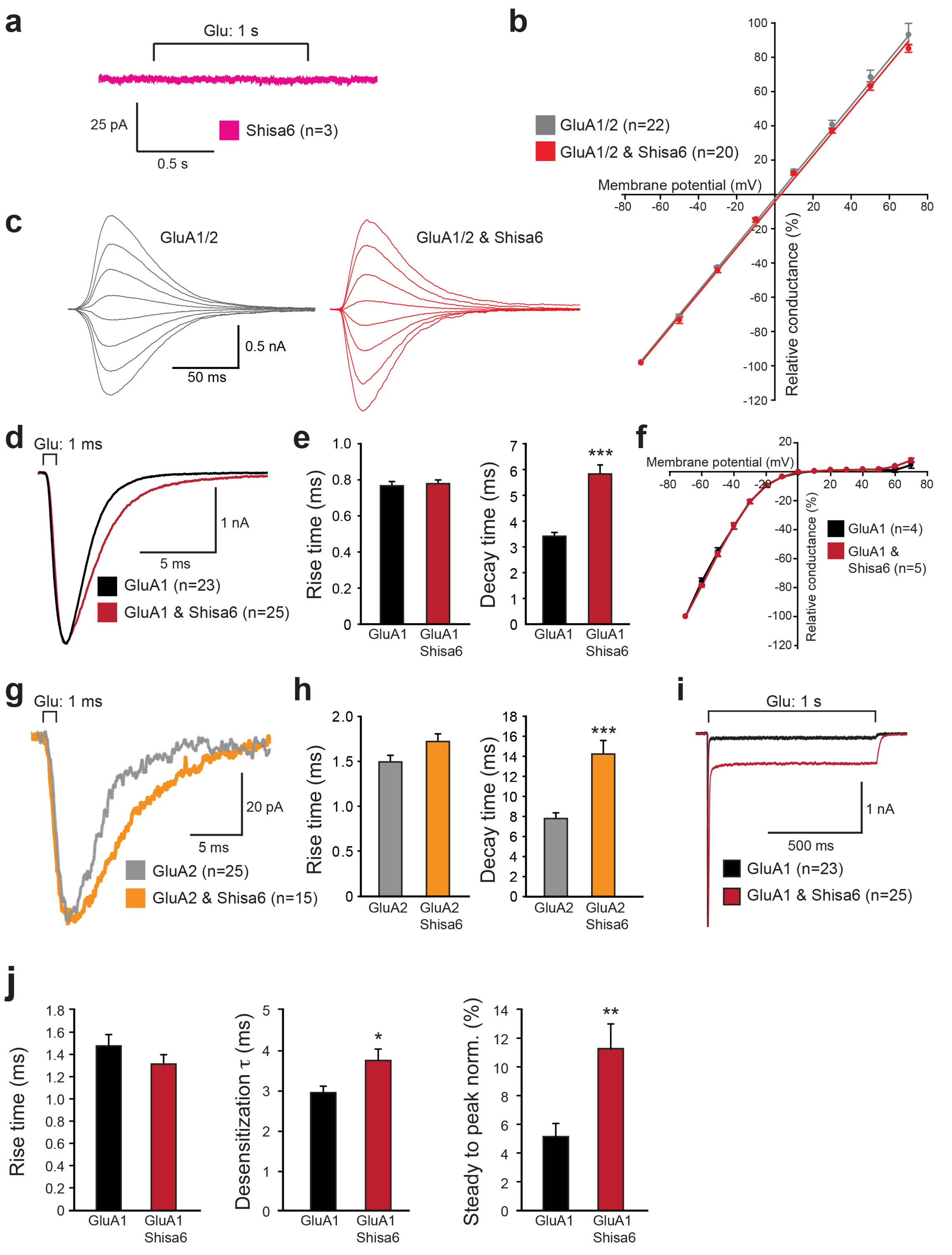

Supplementary Figure 4. Shisa6 modulates the kinetics of heteromeric and monomeric AMPAR currents in HEK293 cells. **a**) Expression of Shisa6 in the absence of an AMPAR (pink; n=3) does not lead to currents when stimulated by 1 s glutamate, indicating that Shisa6 is not a pore-forming

subunit. **b,f**) IV-curve of heteromeric GluA1/2 (b), and homomeric GluA1-containing (f) AMPAR expressed in HEK293 cells in the presence and absence of Shisa6 shows that Shisa6 does not alter rectification of GluA2-containing or GluA2-lacking AMPARs. **c)** Example traces of heteromeric GluA1/2 AMPAR while holding the membrane potential from 70 mV to –70 mV. **d,g,i**) Peak-scaled example traces of whole-cell recording from HEK293 cells expressing homomeric GluA1 (d,i) or GluA2 (g) AMPAR channels in absence (black/gray) or presence (red/orange) of Shisa6. Currents were evoked by direct application of 1 mM glutamate during 1 ms (d,g), or by direct application of 1 mM glutamate during 1 s (i). **e,h**) For the 1-ms glutamate application, bar graphs (mean±SEM) summarize changes of AMPAR currents mediated by homomeric GluA1 (e) or GluA2 (h) AMPARs in HEK293 cells in the presence and absence of Shisa6 in terms of rise time (e, 0.77±0.02 *vs*. 0.78±0.02 ms; *P*=0.708; h, 1.49±0.07 *vs.* 1.74±0.08, *P*=0.051), and decay time (e, 3.41±0.15 *vs*. 5.83±0.29 ms; *P*<0.001; h, 7.94±0.63 *vs*. 14.30±1.35, *P*<0.001). **j**) For the 1-s glutamate application, bar graphs (mean±SEM) summarize changes in rise time (1.48±0.10 *vs.* 1.25±0.09 ms, *P*=0.230), desensitization time constant (2.94±0.16 *vs.* 3.74±0.28, *P*=0.016), and steady-state AMPAR-mediated currents of homomeric AMPARs (5.18±0.91 *vs.* 11.30±9.25 % of peak current, *P*=0.004) in absence (black) or presence (red) of Shisa6. * *P*<0.050, ** *P*<0.010, *** *P*<0.001 (Student's t-test).

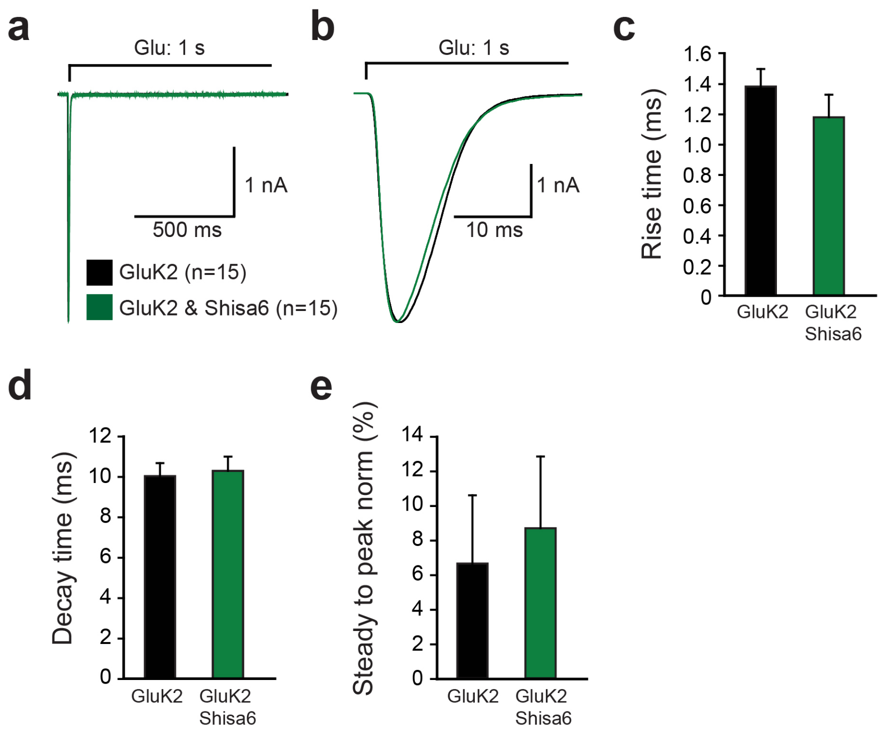

Supplementary Figure 5. Shisa6 does not alter kainate receptor kinetics. **a**) Peak-scaled example traces of whole-cell recordings from HEK293 cells expressing GluK2 (GluR6) homomeric receptors in the absence (black; n=15) or presence (dark green; n=15) of Shisa6. Currents were evoked by direct application of 1 mM glutamate during 1 s. **b**) Zoom-in of panel **a** to visualize changes in kinetics of GluK2 (GluR6). **c–e**) Bar graphs (mean±SEM) summarize changes in rise time (c), decay time (d), and steady-to-peak ratio (e) of currents mediated by homomeric kainate receptors in HEK293 cells in the presence and absence of Shisa6.

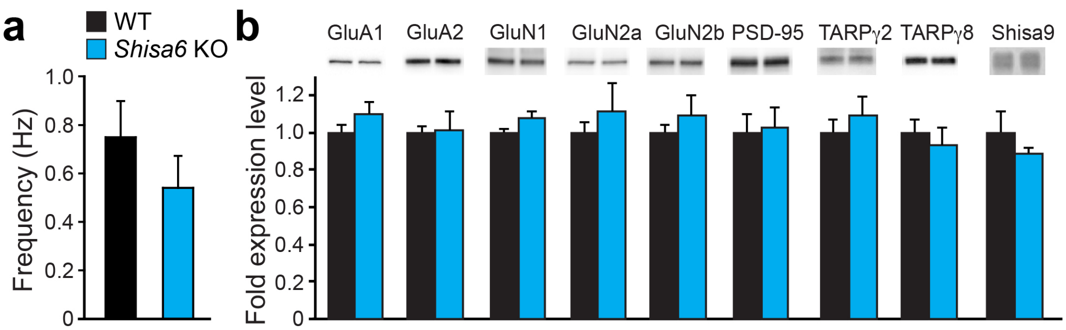

Supplementary Figure 6. Shisa6 deletion does not alter mEPSC frequency or lead to compensatory effects on synaptic AMPAR expression. **a**) Bar graph (mean±SEM) summarize absence of a significant change in mEPSC frequency (0.75±0.15 *vs.* 0.54±0.13 Hz, P=0.290) recorded in CA1 pyramidal neurons in acute hippocampal slices of *Shisa6* KO and WT littermates (n=19 cells per genotype, from 4 WT and 4 KO animals). **b**) Immunoblots from hippocampal synaptic membrane fractions from WT and *Shisa6* KO mice (n=6 each) do not reveal differences in abundance of AMPAR (GluA1, P=0.216; GluA2, P=0.924), NMDAR (GluN1, P=0.101; GluN2a, P=0.565; GluN2b, P=0.481), PSD-95 (P=0.862), TARPs (γ2, P=0.545; γ8, P=0.611) or CKAMP44/Shisa9 (P=0.379), when expressed as fold change over WT samples. Signal was normalized to total protein level.

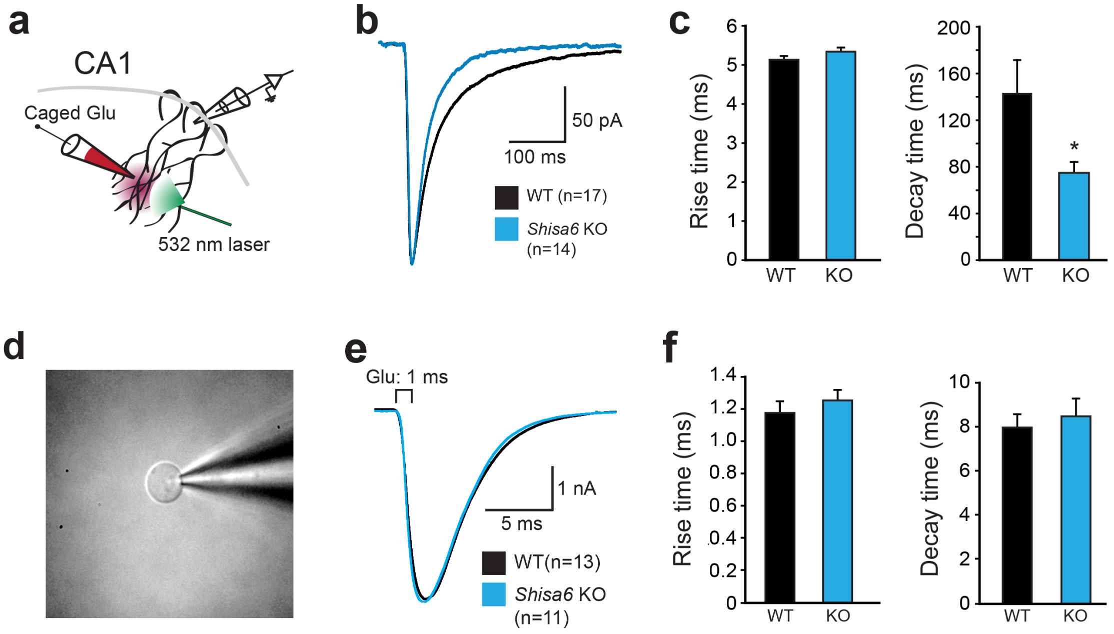

Supplementary Figure 7. Shisa6 affects dendritic AMPAR currents. **a**) Experimental design used for glutamate uncaging. Caged-glutamate (red) was puffed into the slice onto the dendrites of CA1 pyramidal cells. A 532-nm laser (green) was used to uncage glutamate. **b**) Superimposed example recordings from glutamate uncaging experiments of CA1 pyramidal cells of *Shisa6* KO animals (n=14 from 5 animals) and WT littermates (n=17 from 5 animals). Currents were evoked by 10-ms laser uncaging of Rubi-glutamate (1 mM). Light-induced currents were completely abolished by DNQX (10 µM; n=3, not shown). **c**) Bar graphs (mean ± SEM) summarize changes in rise time (WT 5.13±0.096 *vs.* KO 5.34±0.108 ms, *P*=0.170) and decay time (142.67±28.93 ms *vs.* KO, 74.87±9.35 ms, *P*=0.044) of the AMPAR currents. **d**) Picture of a nucleated patch from a CA1 pyramidal cell. **e**) Superimposed example recordings from nucleated patches of CA1 pyramidal cells of *Shisa6* KO animals (n=12 from 3 animals) and WT littermates (n=12 from 5 animals). Currents were evoked by a 1-ms application of 1 mM glutamate. **f**) Bar graphs (mean±SEM) summarize changes in rise time and decay time of nucleated patch recordings (rise time (ms): WT, 1.18±0.72 *vs.* KO 1.26±0.07, *P*=0.8201; decay time (ms): WT, 8.32±0.66 *vs.* KO 8.56±0.72, *P*=0.800). The absence of differences suggests that Shisa6 does not interact with functional AMPARs at the cell soma.

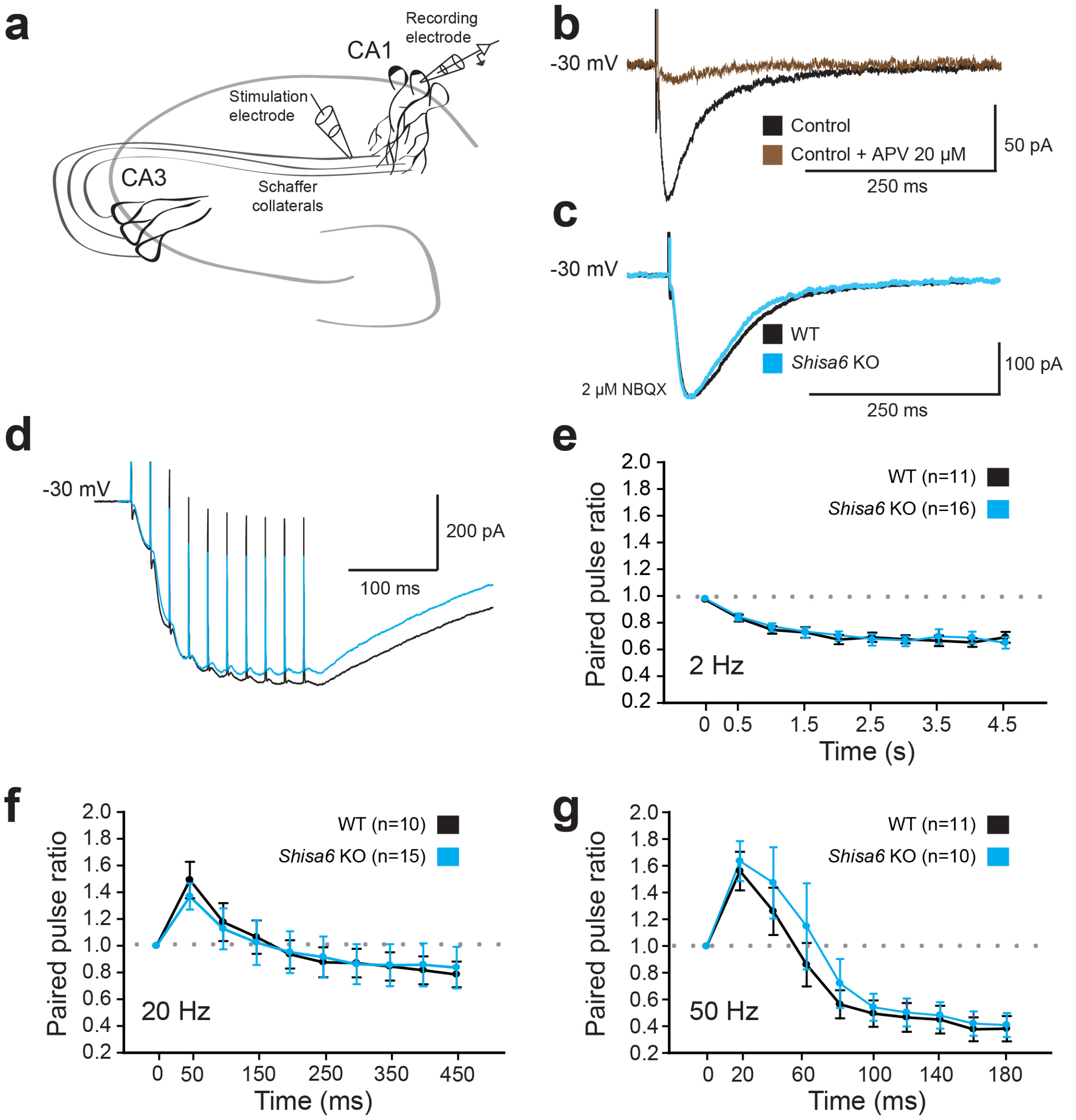

Supplementary Figure 8. Shisa6 has no presynaptic effect as measured by NMDAR currents. **a**) Diagram showing the recording site and stimulation electrode used for stimulation of Schaffer collateral synapses in the presence of NBQX and Gabazine. **b**) Example trace recorded from CA1 pyramidal neurons of WT animals in absence (black) or presence (brown) of 20 μM APV in response to 50-Hz stimulation of synaptic inputs from Schaffer collateral voltage clamped at –30 mV. **c**) Example trace recorded from CA1 pyramidal neurons of WT animals (black) or *Shisa6* KO (blue) in response to 50-Hz stimulation of synaptic inputs from Schaffer collateral voltage clamped at –30 mV, showing that rise and decay times of NMDAR currents, measured in the presence of 2 μM NBQX, are not affected in *Shisa6* KO (P>0.500). **d**) Superimposed example

traces recorded from CA1 pyramidal neurons of *Shisa6* KO (blue) animals and WT littermates (black) in response to 50-Hz stimulation of synaptic inputs from Schaffer collateral voltage clamped at –30 mV. **e–g**) Pulse ratios of electrically-evoked EPSCs (at –30 mV) from CA1 pyramidal neurons of *Shisa6* KO (blue, n=15 from 4 animals) animals and WT littermates (n=12 from 5 animals) at 2 (**e**), 20 (**f**) and 50 (**g**) Hz. Cell numbers used are indicated.

Figure 1b

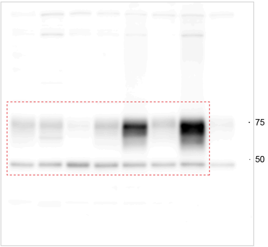

Figure 2d

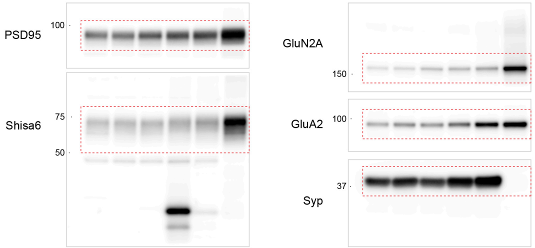

Figure 2e and 3a

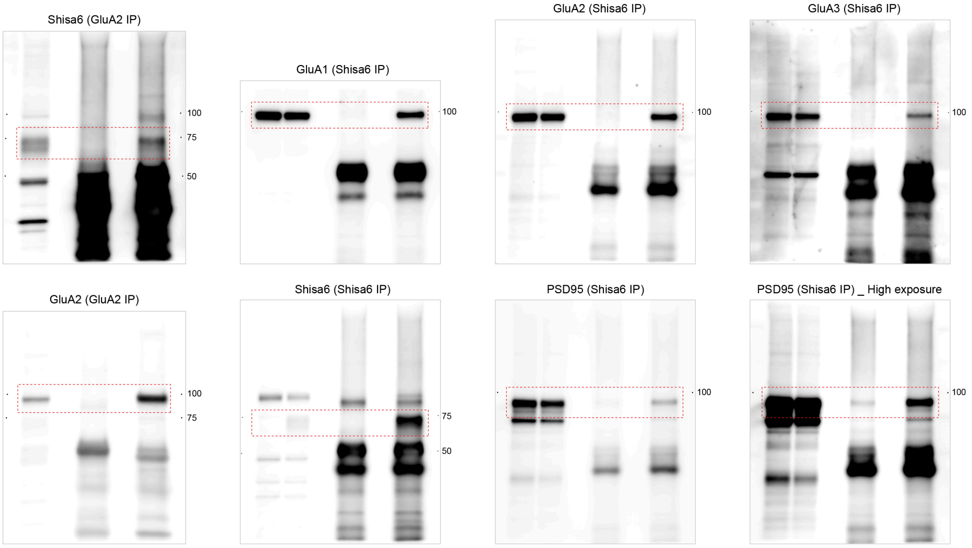

Figure 2f

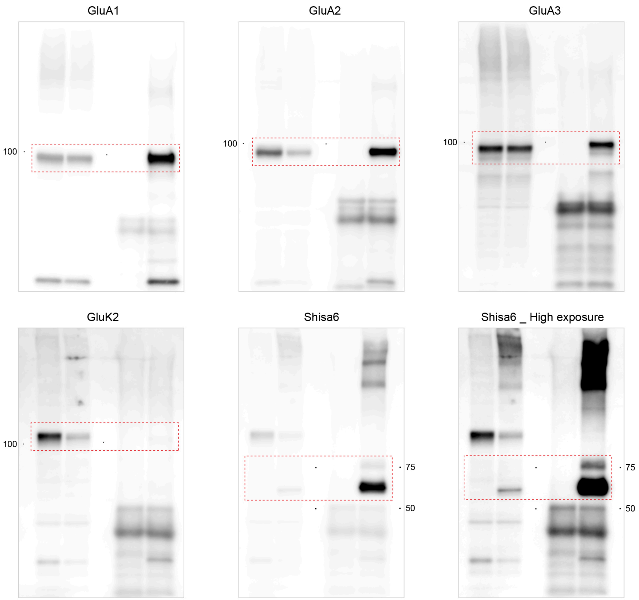

Supplementary Figure 2d

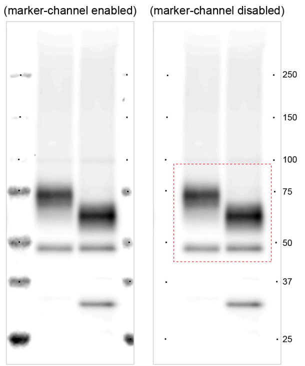

Supplementary Figure 9. Whole immunoblot compilation. Whole immunoblots are presented from which sections are included in figures 1b, 2d, 2e, 2f, 3a, and supplementary figure 2d. Sections are outlined by a red dashed line. Numbers represent apparent molecular weights in kDa. Black dots indicate the center of marker bands, as is demonstrated for supplementary figure 2d.

Gene name	Uniprot recommended name(s)	Uniprot ID	PDZ-domains	Number of unique peptides						Unique peptide spectral-count						Average KO spectral-count	Average WT spectral-count	Average WT/KO spectral-count	Average KO/WT spectral-count	Beta-binomial test (p-value)	Percent coverage
				KO1	KO2	KO3	WT1	WT2	WT3	KO1	KO2	KO3	WT1	WT2	WT3						
Shisa6	Protein shisa-6 homolog	Q3UH99	0	0	0	0	21	23	21	0	0	0	150	136	166	0.0	150.7	N/A	0.0%	<0.0001	47%
	Protein shisa-6 homolog: Exon3-unique peptide SGGPDLHNFISSGFVTLGR	-	-	-	-	-	-	-	-	0	0	0	3	5	5	-	-	-	-	-	-
Gria1	Glutamate receptor 1	P23818	0	4	4	4	14	20	19	4	4	4	37	48	39	4.0	41.3	10.3	9.7%	<0.0001	34%
Gria2	Glutamate receptor 2	P23819	0	11	10	11	27	31	30	12	10	13	77	79	74	11.7	76.7	6.6	15.2%	<0.0001	49%
Gria3	Glutamate receptor 3	Q9Z2W9	0	1	1	0	15	16	15	1	1	0	19	19	17	0.7	18.3	27.5	3.6%	<0.0001	32%
Gria4	Glutamate receptor 4	Q9Z2W8	0	0	0	0	0	1	1	0	0	0	0	1	1	*	*	*	*	*	*
Olfm1	Noelin	O88998	0	0	0	0	2	1	1	0	0	0	2	1	1	0.0	1.3	N/A	0.0%	0.0186	*
Prrt1	Proline-rich transmembrane protein 1; SynDIG4	O35449	0	1	1	0	2	2	3	1	1	0	5	3	7	0.7	5.0	7.5	13.3%	0.0036	12%
Prrt2	Proline-rich transmembrane protein 2	E9PUL5	0	1	2	1	2	1	2	1	3	1	2	1	2	1.7	1.7	1.0	100.0%	0.9992	*
Cacng8	Voltage-dependent calcium channel gamma-8 subunit; TARP gamma-8	Q8VHW2	0	3	2	3	6	7	5	3	3	3	14	12	9	3.0	11.7	3.9	25.7%	0.0008	39%
Dlg1	Disks large homolog 1; SAP97	Q811D0	3	1	0	0	1	1	1	1	0	0	1	1	1	0.3	1.0	3.0	33.3%	0.3063	*
Dlg2	Disks large homolog 2; PSD93	Q91XM9	3	0	0	0	0	2	2	0	0	0	0	2	2	*	*	*	*	*	*
Dlg3	Disks large homolog 3; SAP102	P70175	3	0	0	1	4	8	5	0	0	1	4	8	5	0.3	5.7	17.0	5.9%	0.0011	14%
Dlg4	Disks large homolog 4; PSD95	Q62108	3	1	0	2	13	9	12	1	0	2	15	13	20	1.0	16.0	16.0	6.3%	<0.0001	35%
Lrrc7	Leucine-rich repeat-containing protein 7; Densin-180	Q80TE7	1	0	0	0	8	17	8	0	0	0	9	18	8	0.0	11.7	N/A	0.0%	0.0002	19%
Mpp2	MAGUK p55 subfamily member 2	Q9WV34	1	0	0	0	1	0	1	0	0	0	1	0	1	*	*	*	*	*	*
Mpp5	MAGUK p55 subfamily member 5	Q9JLB2	1	0	0	0	0	1	1	0	0	0	0	1	1	*	*	*	*	*	*
Magi2	Membrane-associated guanylate kinase, WW and PDZ domain-containing protein 2	Q9WVQ1	6	23	28	26	17	21	25	31	48	36	24	27	30	38.3	27.0	0.7	142.0%	*	*
Magi3	Membrane-associated guanylate kinase, WW and PDZ domain-containing protein 3	Q9EQJ9	6	0	0	0	1	1	0	0	0	0	1	1	0	*	*	*	*	*	*
Mpdz	Multiple PDZ domain protein	Q8VBX6	13	0	0	0	0	1	1	0	0	0	0	1	1	*	*	*	*	*	*
Nos1	Nitric oxide synthase, brain	Q9Z0J4	1	31	35	32	32	29	32	48	52	47	41	37	42	49.0	40.0	0.8	122.5%	*	*
Cask	Peripheral plasma membrane protein CASK	O70589	1	0	2	0	3	1	1	0	2	0	3	1	1	0.7	1.7	2.5	40.0%	0.2493	*
Lin7a	Protein lin-7 homolog A	Q8JZS0	1	1	0	1	2	2	1	1	0	1	2	2	1	0.7	1.7	2.5	40.0%	0.2500	*
Pclo	Protein piccolo	Q9QYX7	1	1	2	1	1	0	1	2	3	2	1	0	2	*	*	*	*	*	*
Rapgef2	Rap guanine nucleotide exchange factor 2	Q8CHG7	1	2	2	1	3	1	2	2	2	1	3	1	2	1.7	2.0	1.2	83.3%	0.7638	*
Myo18a	Unconventional myosin-XVIIIa	Q9JMH9	1	80	85	86	76	82	76	150	190	165	151	142	153	168.3	148.7	0.9	113.2%	*	*

Supplementary Table 1. Mass spectrometric analysis of native hippocampal Shisa6 complexes reveals association with PDZ domain-containing proteins and established AMPAR interactors. This analysis of native Shisa6 complexes, immunoprecipitated from the hippocampi of *Shisa6* WT and KO animals (crude synaptic membranes; n=3 IPs per genotype), identified PSD-95 (Dlg4) as most prominent PDZ domain-containing interactor. IP-ed proteins: Shisa6 (dark blue), AMPAR subunits (light blue), established non-PDZ AMPAR interacting proteins[9,10] (dark yellow), PDZ-containing interactors (light yellow). Selection criteria were: 1) For relative protein quantification, all WT samples should have ≥1 unique peptide found. 2) The average WT/KO spectral-count value should be ≥1 (or N/A), indicating enrichment in the WT samples. 3) Performing a beta-binomial test, enrichment in the WT samples should reach $P \leq 0.01$ (green). Failing a particular selection criterion resulted in an exclusion marker (*). Percent coverage, percent of the database protein sequence covered by all matching peptides (<30%, light orange; ≥30%, orange). Note the identification of the Shisa6 exon-3 unique peptide (SGGPDLHNFISSGFVTLGR).

Gene	Purpose	Forward	Sequence forward primer	Reverse	Sequence reverse primer	Amplicon size (nts)
Shisa6	PCR	Ex1F	CTTCACCGTCTACATCACTTG	Ex6R	CGTGGAGACAAGACCCTGTC	753 (incl exon 3)<br>657(exl exon 3)
Shisa6-WT	PCR	RZ 742	CCGGAGAAGGACAAGACCAACTTCAC	RZ 804R	GATGGCCTGAACATTACAGAAGCATGAACTC	767
Shisa6-KO	PCR	RZ 803	CTGACCGGTCCGTCTCGCCTTTC	RZ 804R	GATGGCCTGAACATTACAGAAGCATGAACTC	515
Shisa6	RT-PCR	F1	GCCAGCGCGACTGTTACTTAC	R1	TCGCTGTTGTTGCACTCGA	89
Gapdh	RT-PCR	F1	TGCACCACCAACTGCTTAGC	R1	GGCATGGACTGTGGTCATGA	87
β-actin	RT-PCR	F1	GCTCCTCCTGAGCGCAAG	R1	CATCTGCTGGAAGGTGGACA	61
HPRT	RT-PCR	F1	ATGGGAGGCCATCACATTGT	R1	ATGTAATCCAGCAGGTCAGCAA	56

Supplementary Table 2. Sequence of DNA primers used. For PCR experiments to detect the presence or absence of exon 3 in Shisa6 or the WT/KO allele of *Shisa6*, as well as for real-time PCR, and for detection of the *Shisa6* genotype in animals and neurons, different primer sets were used. The forward and reverse primer, as well as the size of the amplicon generated, are indicated.

Experiment	Shisa6	CKAMP44/ Shisa9	Methods Shisa6	Methods CKAMP44/Shisa9
GluA expression	Not affected	Enhanced	Acute slice (CA1), Immunoblotting	Acute slice (DG), Outside-out patch (DG), GluA1 cluster analysis
Rise time	Slowed	Not affected	Acute slice (CA1)	Acute slice (DG)
Deactivation	Slowed	Slowed	HEK293, Acute slice (CA1), Dendritic glutamate uncaging (CA1)	Outside-out patch (DG)
Desensitization	Slowed	Faster	HEK293	HEK293, Outside-out patch (DG)
Steady-state conductance	Enhanced	Reduced	HEK293	HEK293, Outside-out patch (DG)
Recovery from desensitization	Slowed	Slowed	HEK293	Outside-out patch (DG)
mEPSC frequency	Not affected	Enhanced	Acute slice (CA1)	Acute slice (DG)
Synaptic depression	Reduced	Enhanced	Acute slice (CA1)	Acute slice (DG)

Supplementary Table 3. Comparison of functional significance of Shisa proteins. Actions of Shisa proteins expressed in heterologous systems, and as defined by virtue of gene deletion in slices of CA1 (*Shisa6*) or DG (*CKAMP44/Shisa9*[11]).

Supplementary methods

Protein domain prediction and sequence alignment

The signal sequence was predicted using the online tool SequenceP4.1 (http://www.cbs.dtu.dk/services/SignalP/)[12]. The predicted molecular weight was determined using the online tools of Expasy (http://web.expasy.org/compute_pi/) and EnCor Biotechnology (http://www.encorbio.com/protocols/Prot-MW.htm). Sequence alignments were generated with CLC workbench 3.0.

(Real-Time) Polymerase Chain Reaction

Primers – Primers for PCR and real-time PCR were generated using Primer3.0. The final sets of primers are listed in Supplementary Table 2.

RNA isolation and cDNA synthesis – RNA from several tissues was extracted as previously described[13]. Samples were DNase-I treated according to the manufacturer's instructions (20 U per µg RNA; Boehringer) to remove traces of genomic DNA, which was verified by using intron-specific PCR primers (data not shown). RNA concentration was determined using the NanoDrop ND-1000 spectrophotometer (NanoDrop Technologies), and the integrity of RNA was checked by gel electrophoresis (1%-TBE-agarose gel). Random-primed (25 pmol; Eurofins MWG Operon) cDNA synthesis was performed on individual RNA samples (~1 µg total RNA).

PCR for exon 3 – PCR products generated were visualized on a 1.75 % TAE gel (Supplementary Fig. 1), and sequenced using the forward primer (Supplementary Table 2) in quadruplicate.

Real-time qPCR – Real-time qPCR reactions (20 µL; ABI PRISM 7700) on pooled tissue (3 mice) were performed using a 96-well format with transcript-specific primers (300 nM) on cDNA corresponding to ~20 ng RNA and SYBR Green reagents (Applied Biosystems)[13]. Only primer sets (Eurofins MWG Operon) with proper amplification efficiency[13,14] were used. Cycle threshold (Ct) values were used to calculate the relative level of gene expression, where Ct value is the fractional cycle number at which the fluorescent signal of a reaction passes the threshold (reaching intensity above background). Expression level of three housekeeping genes (GAPDH, β-actin, HPRT) was measured as reference for input. Expression is denoted using normalized Ct values on a $\log_2$-scale. Let normalized Ct-values be denoted by $Ct\text{norm}_x$ (where x represents *Shisa6* expression, y represents the geometric mean of Ct-values of the housekeeping

genes, and *i* represents a given sample), $Ct\text{norm}_{xi}$ then is given by $Ct\text{norm}_{xi} = Ct_{xi} - Ct_{yi}$. As a bigger Ct-value correlates with a lower expression level, for practical purposes, $Ct\text{norm}_{xi}$ values were converted into con$Ct\text{norm}_{xi}$ values, calculated as con$Ct\text{norm}_{xi} = -Ct\text{norm}_{xi} + 15$. Due to this conversion, the final positive value of *Ct* is positively correlated with relative gene expression level, which makes the visualization simpler. Relative gene expression levels were expressed as conCt*norm*-values ±SEM.

DNA expression constructs

pTRCGw-IRES2-EGFP is an adapted version of the pTRCGw plasmid (5.6 kb), which was a kind gift from Dr. J. Verhaagen (The Netherlands Institute for Neuroscience, Amsterdam, The Netherlands), carrying the ampicillin resistance gene, inverted terminal repeats (ITR), human immediate early cytomegalovirus promoter (CMV), wood chuck posttranscriptional regulatory element (WPRE) and multiple restriction sites. pTRCGw-IRES2-EGFP was created by insertion of a Gateway-cloning pDEST recombination site (Invitrogen) and pIRES-EGFP element, upstream of the existing WPRE and pA modules. The full-length coding DNA for exon3-containing mouse Shisa6 was obtained from Genscript as a pENTR4 construct (reference NM_001034874.3 / NP_001030046.1 + exon3 sequence), and subsequently Gateway-cloned into pTRCGw-IRES2-EGFP, yielding the Shisa6-pTRCGw-IRES2-EGFP construct. This plasmid was modified to FLAG-Shisa6-pTRCGw-IRES2-EGFP by PCR-mediated insertion of a tandem FLAG-tag (sense 5'-GGT GAT TAT AAA GAT CAT GAT ATC GAT TAC AAG GAT GAC GAT GAC AAG CAC-3', corresponding peptide: GDYKDHDIDYKDDDDKH) between codon 36 (GGG, Glycine) and codon 37 (AAC, Asparagine) of the Shisa6 cDNA.

For HEK293 cell expression, GluA1 isoform 1 and GluA2 isoform 1 were Gateway-cloned into the pTRCGw-IRES2-EGFP vector, yielding respectively GluA1-pTRCGw-IRES2-EGFP and GluA2-pTRCGw-IRES2-EGFP. GluA3 was cloned into the pRK5 vector. GluK2(Q) was cloned into the pcDNA3 vector (Invitrogen) as described previously[15].

For expression in hippocampal neuronal cultures, N-terminally Flag-tagged Shisa6 was subcloned in the pBI Tet-On vector (Clontech). cDNA corresponding to the GluA subunits was as described previously[16]. Homer 1C::EGFP and Homer 1C::DsRed were generated by subcloning Homer 1C cDNA into the pcDNA3 vector (Invitrogen).

For FLIM experiments, eGFP was inserted at position 253 of PSD-95 as previously described[17]. The mCherry tag was inserted 21 amino acids before the stop codon of Shisa6. All DNA constructs were sequence verified before use.

Generation of *Shisa6* KO mice

The mouse chromosome 11 sequence (nucleotide # 66,299,000~66,379,000) was retrieved from the Ensembl database and used as reference (Caliper Life Sciences). The mouse RP23-26O19 BAC DNA was used for generating the homology arms and conditional region for the gene targeting vector, The 5'-homology arm (~3.0 kb), 3'-homology arm (~4.0 kb), and conditional region (~1.0 kb) were generated by PCR and fragments were cloned in the LoxFtNwCD vector. The final vector contained loxP sequences flanking the conditional KO region (~1.0 kb), the Neo expression cassette (for positive selection of the ES cells) flanked by FRT sequences (for the subsequent removal of the Neo cassette), and a DTA expression cassette (for negative selection of the potentially targeted ES cells). The final vector was confirmed by both restriction endonuclease digestion (BamHI) and by end sequencing analysis. SwaI was used for linearizing the final vector prior to electroporation, and 30 µg of NotI-linearized final KO vector DNA was electroporated into ~10^7 C57BL/6 ES cells and selected with 200 µg per mL G418. From 192 ES clones selected for PCR based screening, two targeted clones were confirmed to be correctly targeted and have a single Neo insertion. Blastocyst injection resulted in chimeric offspring generating *Shisa6*$^{tm1a1(Caliper)CNCR\text{-}VUA}$ mice. These mice were backcrossed with FLP-mice to get rid off the Neo insertion to generate the *Shisa6*$^{tm1a2(Caliper)CNCR\text{-}VUA}$ line, the latter of which is currently kept at the VU University. For the purpose of this study, the *Shisa6*$^{tm1a2(Caliper)CNCR\text{-}VUA}$ line was crossed with Cre-mice to generate full *Shisa6* KO mice, officially named *Shisa6*$^{tm1b(Caliper)CNCR\text{-}VUA}$, in which exon 1 was deleted. For this study, *Shisa6* KO animals were derived from heterozygous crossing, except for culturing experiments, in which homozygous mice were used. In all cases, the genotype was established by PCR on genomic DNA (Supplementary Table 2).

Antibodies

Anti-Shisa6 antibody was raised in rabbit against sequence DRYRMTKMHSHPSA (position 494-507 in Shisa6) (Genscript). The antibody was affinity-purified against the antigenic peptide, suspended at 1 mg per mL in PBS containing 0.02% NaN3, and stored at –20 °C. Antibodies used for immunoblotting were anti-Shisa6 (see above;

1:1,000), anti-GluA1 (Abcam, ab109450, 1:20,000), anti-GluA2 (Neuromab clone L21/32, 1:1,000), anti-GluA3 (Abcam, ab40845, 1:500), anti-GluK2 (Santa Cruz, C-18, 1:1,000), anti-GluN2A (Abcam, ab14596, 1:2,000), anti-PSD-95 (Neuromab clone K28/43, 1:50,000), and anti-Synaptophysin (Genscript A01307, 1:2,000). Antibodies used in immunoprecipitation were anti-Shisa6 (see above), anti-GluA2 (Neuromab clone L21/32), anti-FLAG M2 (Sigma, F1804) and IgG control (Genscript, whole protein, A01007). Antibody used for QD tracking was GluA2 (MAB397; Millipore). Antibodies used for immunofluorescence cytochemistry were mouse IgG1 anti Flag M2 (Sigma, F3165, diluted 1:1,000), mouse IgG2b anti GluA2 extracellular domain (gift from E. Gouaux, OHSU, Portland OR, used at 2 μg per mL), mouse IgG2a and anti PSD-95 (NeuroMab clone K28/43, diluted 1:500). Alexa-conjugated isotype-specific secondary antibodies were obtained from Invitrogen. See below for more information on immunocyto- and immunohistochemistry.

Subcellular fractionation

Subcellular fractions were prepared as described previously[9,18] with some modifications. All steps were performed at 4 °C and in the presence of EDTA-free Complete protease inhibitor (Roche). Hippocampal tissue was homogenized in buffer containing 0.32 M Sucrose and 5 mM HEPES, pH 7.4 (Homogenate fraction), and centrifuged at 1,000x *g* for 10 minutes. The supernatant was either (1) centrifuged at 18000x *g* for 30 minutes, yielding a crude synaptic membrane pellet (P2 fraction), (2) centrifuged at 120,000x *g* for 2 h, yielding the crude synaptic membrane + microsome pellet (P2+M fraction), or (3) layered onto a discontinues 0.85 / 1.2 M sucrose density gradient and centrifuged at 120,000x *g* for 2 h. The 0.85 / 1.2 M sucrose interface was collected as the synaptosome fraction. Synaptosomes were hypotonically shocked by dilution in 5 mM HEPES pH 7.4 and subjected to a second round of centrifugation on a 0.85 / 1.2 M sucrose density gradient. The synaptic membrane fraction was collected at the 0.85 / 1.2 M sucrose interface. Alternatively, synaptosomes were mixed with Triton X-100 (0.32 M sucrose and 5 mM HEPES, pH 7.4) to a final concentration of 1%, incubated for 1 h, and layered on top of a 1.0 / 1.5 / 2.0 M sucrose density gradient. After 2 h centrifugation 120000x *g*, the preliminary PSD fraction was collected at the 1.5 / 2.0 M interface. A second 1% Triton X-100 extraction and subsequent 1.5 / 2.0 M sucrose density gradient purification yielded the final PSD fraction.

Immunoprecipitation of Shisa6 and GluA2 protein-complexes from mouse hippocampus

All subsequent steps, excluding protein-elution, were performed at 4 °C. Hippocampal tissue from WT and *Shisa6* KO mice was homogenized in buffer A (0.32 M sucrose, 10 mM HEPES pH 7.4, and EDTA-free Complete protease inhibitor) and centrifuged at 1,000x *g* for 10 minutes. Centrifuging the supernatant at 18,000x *g* for 30 minutes yielded the crude synaptic membrane pellet. This pellet was resuspended to 5 mg protein per mL in buffer B (1% DDM, 150 mM NaCl, 25 mM HEPES, and EDTA-free complete protease inhibitor), incubated for 1 h while mixing gently, and centrifuged at 20,000x *g* for 20 minutes. This extraction-procedure was repeated once more on the remaining pellet. The supernatant from both extractions was pooled and subjected to a second round of centrifugation at 20,000x *g* for 20 minutes. Antibody (Anti-Shisa6 antibody, anti-GluA2 antibody, or IgG control) was added to the supernatant of the DDM-extracted crude synaptic membrane fraction, incubated O/N, and immobilized to Protein A/G agarose beads (Santa Cruz). The agarose beads were washed 4 times with buffer C (0.1% DDM, 150 mM NaCl, 25 mM HEPES pH7.4) and bound proteins were eluted by incubation with Laemmli sample buffer.

In-gel Tryptic digestion

The free cysteine residues of *Laemmli* buffer-eluted proteins were blocked by addition of acrylamide (3.75% final concentration) for 30 minutes at room temperature. Proteins were resolved on a 10% SDS polyacrylamide gel, fixed, and stained with colloidal Coomassie Blue G-250. Sample lanes were cut into 3 segments, destained, and the proteins in-gel digested with Trypsin/Lys-C mix (Promega) during overnight incubation at 37 °C. The peptides were extracted twice with 50% acetonitrile + 0.1% trifluoroacetic acid for 40 minutes, once with 80% acetonitril + 0.1% trifluoroacetic acid for 20 minutes, dried by speedvac, and stored at –80 °C.

HPLC-MS-MS

Peptide samples were re-dissolved in 0.1% acetic acid and loaded onto a PepMap100 C18 precolumn (300 µm i.d., 5 µm particle size; Dionex) connected to an UltiMate 3000 HPLC system (Dionex). Separation was achieved on a 200 mm Alltima C18 in-house packed column (100 µm i.d., 3 µm particle size) by using an aquous-organic gradient of 5% to 40% acetonitrile + 0.1% formic acid for 27 minutes at a flow rate of 400 nL per min.

Eluates were electrosprayed directly into a TripleTOF 5600+ system (Absciex) operated in Information Dependent Acquisition mode. One full-scan cycle consisted of a precursor ion scan (m/z range of 350 to 1250, charge state +2 to +5, 90 cps intensity threshold, and 0.25 s accumulation time) followed by MS-MS analysis of the top 20 most abundant ions (m/z range 200 to 1800, 0.09 seconds accumulation time). Former target ions were excluded for 10 s. Each individual immunoprecipitation experiment was measured once.

MS data analysis

TripleTOF 5600+ output files were converted from wiff to mzML (centroid) format using the AB SCIEX MS Data Converter utility and searched against the mouse UniProtKB/Swiss-Prot canonical sequence database (version 2014-03-21) by means of the Morpheus search engine (version 1.0.0.0) [18,19]. Methionine oxidation and protein N-terminal acetylation were selected as variable modifications, and proprionamide was set as fixed cysteine modification. The maximum mass tolerance for precursor and product ions was 25 ppm and 0.025 Da respectively. Trypsin (no proline rule) was selected as protease, allowing up to one missed cleavage. A target-decoy database was created on the fly and the maximum Peptide Spectrum Match (PSM) false discovery rate (FDR) was set to 1%. An in-house developed script was used to import the PSM results from the Morpheus pepXML files into ProteinProphet[19,20], where the final protein inference and protein validation was performed (protein FDR of 1%). The summed spectral count value of all unique peptides was used for relative protein quantification (peptides shared within or between groups were omitted). The statistical significance of the results was analyzed using a non-normalized beta-binomial test[20], a method developed specifically for the analysis of spectral count-based datasets, where results do not follow a normal distribution.

Immunocytochemistry of cell cultures

To study the surface localization of Shisa6, independent of the in-house antibody against Shisa6, overexpression of Shisa6 was performed in neuronal cultures. To minimize the over-expression level of Flag-Shisa6 and yet observe its localization in mature neurons, Flag-Shisa6 was expressed in *Shisa6* KO neurons using an inducible promoter. This enables transfection of neurons at DIV6, and to induce expression of Flag-Shisa6 by addition of doxycycline to the culture medium until DIV16, and then observe Flag-Shisa6 localization after only a few hours of expression in the *Shisa6* KO background. To this

end, *Shisa6* KO cultured neurons were transfected using Effectene Transfection Reagent (Qiagen) at DIV6 with plasmids of the Tet-On® Gene Expression System (Clontech). In brief, two plasmids were transfected, one encoding a tetracycline-binding transactivator protein, and the second response plasmid encoding Flag-tagged Shisa6 under the control of a Tetracycline-response element (TRE). At DIV15, expression of Flag-Shisa6 was induced by addition of doxycycline to the culture medium (2 µg per mL). Doxycycline-bound transactivator then binds the TRE in the response plasmid to activate transcription of Flag-Shisa6. To localize surface-expressed Shisa6 relative to synaptic markers after 18 h of induced expression, cells were live-stained for surface Flag and surface GluA2. First, 10 mM HEPES was added to the culture dish to maintain pH during live staining and all steps were carried out at 37 °C. Antibodies were diluted in culture medium containing 1% BSA (Sigma A3059). Coverslips were incubated on drops of culture medium containing BSA for 2 minutes to minimize non-specific binding, moved to droplets of medium containing antibody for 6 minutes, then rinsed once in culture medium and placed in warmed fixative containing 4% PFA and 4% sucrose in PBS for 15 minutes at RT. All subsequent steps were performed at RT.

Fixed coverslips were rinsed in PBS and aldehydes were quenched with 50 mM NH_4Cl in PBS for 10 minutes followed by additional PBS rinses. To access PSD-95, cells were permeabilized with 0.2% Triton X100 (Sigma T9284) in PBS for 5 minutes, then rinsed with PBS. Non-specific binding was blocked by a 45-minute incubation with PBS containing 2% BSA. PSD-95 antibody was diluted in this blocking solution and incubated with the coverslips for 60 minutes after which coverslips were rinsed and blocked again for 30 minutes prior to incubation with secondary antibodies. Labeled coverslips were mounted on glass slides in ProLong Diamond Antifade Mountant (LifeTechnologies). Images were collected on an upright Leica DM5000 using epifluorescence and a 40x oil objective with LED light source.

Immunohistochemistry of brain slices

We used the method of Yoneyama et al. (2004)[21] In brief, 6-week-old mice were killed by decapitation after anesthesia with isoflurane (4 minutes, 5% isoflurane per 1.5 L air), brains were dissected and quickly frozen. Sections were cut on a cryostat (15 µm) and fixed in Carnoy's solution containing 6:1 ethanol and acetic acid for 10 minutes at 4 °C. The sections were incubated 1 h in blocking buffer containing 4% normal goat serum and 0.3% Triton X-100 in PBS, before they were incubated with first antibodies (anti-

rabbit Shisa6 1:1,000, anti-mouse PSD-95 (Antibodies Inc. Davis, clone K28/43) 1:1,000, and anti-mouse GluA2 (Merck, MAB397) 1:1,000 in blocking buffer (1% normal goat serum and 0.1% Triton X-100 in PBS) at 4 °C during 2 days.

For single labeling, sections were rinsed before being incubated with donkey anti-rabbit antibody coupled to biotin (Life Technologies) 1:200 in blocking solution for 1 h at RT. The sections were further rinsed and incubated in a solution of avidin and biotinylated at RT for 2 h (ABC kit; Vector Laboratories), rinsed and incubated in a solution of streptavidin- Alexa 488 (Life Technologies) 1:800 during 1 h. After rinsing in PBS, sections were mounted between slide and cover slip with a mounting medium containing DAPI (Vectashield Vector Laboratories). For double labeling, sections were rinsed before they were incubated with donkey anti-rabbit Alexa 488 (Life Technologies) and incubated with donkey anti-mouse Alexa 568 (Life Technologies) 1:1,000 in blocking solution for 24 h at 4 °C.

Electrophysiological Recordings

Recordings from HEK293 cells: All electrophysiological recordings we made using a 700B amplifier controlled by PClamp 9.0 in combination with a 1322 digitizer (all from Molecular Devices). Cells were identified and patched using an Olympus BX50WI DIC microscope with a 40x, 0.8 nA W objective (all Olympus cooporation). Data was acquired at a sample frequency of 20 kHz and filtered with an internal 4 pole Bessel filter at 3 kHz. Electrical stimulation was done using a Master 8 and isoflex stimulator (A.M.P.I.).

HEK293 cells were perfused with 32 °C standard artificial CSF (aCSF) during the recordings containing (in mM): 126 NaCl, 3 KCl, 10 D-glucose, 26 $NaHCO_3$, 1.2 NaH_2PO4, 2 $CaCl_2$ and 1 $MgSO_4$, carboxygenated with 95% O_2 and 5% CO_2 to obtain pH7.4 and an osmolarity of 300. HEK293 cells were recorded using borosilicate electrodes (OD 1.5 mm, ID 0.86 mm; Harvard Apparatus) of 2.5–4 MΩ resistance and filled with intracellular solution containing (in mM): 70 Cs-Gluconate, 70 $CsCl_2$, 10 HEPES, 0.1 Spermine, 0.5 NaGTP, 5 Mg_2ATP, 10 EGTA (pH 7.3, 290 osm). HEK 293 cells were gently lifted from the cover slip and placed in front of a Piëzo-driven theta-barrel electrode (TGC 200; Harvard Apparatus), filled with standard aCSF on one side and standard aCSF supplemented with 1 mM L-glutamate Hydrochloride (Sigma Aldrich) on the other side[22]. The experimenter was blind for the constructs used, which was revealed after analysis. Access resistance in HEK cells was typically below 8 MΩ. Cells

with access resistance above 10 MΩ or with current amplitude below 20 pA were excluded from analysis.

Nucleated patches / glutamate uncaging: CA1 pyramidal cells (2–3 week old male mice) were patched using electrodes of 2.5–4 MΩ resistance. The intracellular solution contained (in mM): 70 Cs-Gluconate, 70 $CsCl_2$, 10 HEPES, 0.5 NaGTP, 5 Mg_2-ATP, 10 EGTA and 10 K-Phosphocreatine (pH 7.3, 290 osm). In glutamate uncaging experiments the same intracellular solution was supplemented with 2 μM Alexa 488 (Life Technologies) and 4% Biocytin (Molekula GmbH) for cellular identification. Glutamate was applied to nucleated patches[23] and HEK293 cells with a Piezo-driven double-barrelled pipette as described above. In the glutamate uncaging experiments 1 mM RuBi-Glutamate (Abcam) was continuously puffed by a light protected glass electrode of 2.5–3.5 ΩM resistance into the close proximity of the apical dendrite for the duration of 4 seconds. At 2.5 s into the glutamate puff, glutamate was uncaged using a 50 μm multimode fiber (Thorlabs) close to the application electrode. A 10-ms laser pulse (532 nm wavelength; ML III - 532, ~ 300W;CNI) was used to uncage glutamate. Glutamate uncaging experiments were performed in the presence of 1 μM TTX and 10 μM SR-95531.

Supplementary references

1. Kalyoncu, S., Keskin, O. & Gursoy, A. Interaction prediction and classification of PDZ domains. *BMC Bioinformatics* **11,** 357 (2010).
2. Pei, J. & Grishin, N. V. Unexpected diversity in Shisa-like proteins suggests the importance of their roles as transmembrane adaptors. *Cell. Signal.* **24,** 758–769 (2012).
3. McDonald, N. Q. *et al.* New protein fold revealed by a 2.3-A resolution crystal structure of nerve growth factor. *Nature* **354,** 411–414 (1991).
4. Holland, D. R., Cousens, L. S., Meng, W. & Matthews, B. W. Nerve growth factor in different crystal forms displays structural flexibility and reveals zinc binding sites. *J. Mol. Biol.* **239,** 385–400 (1994).
5. Pallaghy, P. K., Nielsen, K. J., Craik, D. J. & Norton, R. S. A common structural motif incorporating a cystine knot and a triple-stranded beta-sheet in toxic and inhibitory polypeptides. *Protein Sci.* **3,** 1833–1839 (1994).
6. Craik, D. J., Daly, N. L., Bond, T. & Waine, C. Plant cyclotides: A unique family of cyclic and knotted proteins that defines the cyclic cystine knot structural motif. *J. Mol. Biol.* **294,** 1327–1336 (1999).
7. Diez-Roux, G. *et al.* A high-resolution anatomical atlas of the transcriptome in the mouse embryo. *PLoS Biol.* **9,** e1000582 (2011).
8. Schilling, K. & Oberdick, J. The treasury of the commons: making use of public gene expression resources to better characterize the molecular diversity of inhibitory interneurons in the cerebellar cortex. *Cerebellum* **8,** 477–489 (2009).
9. Karataeva, A. R. *et al.* C-terminal interactors of the AMPA receptor auxiliary subunit Shisa9. *PLoS ONE* **9,** e87360 (2014).
10. Schwenk, J. *et al.* High-resolution proteomics unravel architecture and molecular diversity of native AMPA receptor complexes. *Neuron* **74,** 621–633 (2012).
11. Khodosevich, K. *et al.* Coexpressed auxiliary subunits exhibit distinct modulatory profiles on AMPA receptor function. *Neuron* **83,** 601–615 (2014).
12. Petersen, T. N., Brunak, S., Heijne, von, G. & Nielsen, H. SignalP 4.0: discriminating signal peptides from transmembrane regions. *Nat. Methods* **8,** 785–786 (2011).
13. Spijker, S. *et al.* Morphine exposure and abstinence define specific stages of gene expression in the rat nucleus accumbens. *FASEB J.* **18,** 848–850 (2004).
14. Jacobs, E. H. *et al.* Active heroin administration induces specific genomic responses in the nucleus accumbens shell. *FASEB J.* **16,** 1961–1963 (2002).
15. Jaskolski, F. *et al.* Subunit composition and alternative splicing regulate membrane delivery of kainate receptors. *J. Neurosci.* **24,** 2506–2515 (2004).
16. Bats, C., Groc, L. & Choquet, D. The interaction between Stargazin and PSD-95 regulates AMPA receptor surface trafficking. *Neuron* **53,** 719–734 (2007).
17. Sainlos, M. *et al.* Biomimetic divalent ligands for the acute disruption of synaptic AMPAR stabilization. *Nat. Chem. Biol.* **7,** 81–91 (2011).
18. Wenger, C. D. & Coon, J. J. A proteomics search algorithm specifically designed for high-resolution tandem mass spectra. *J. Proteome Res.* **12,** 1377–1386 (2013).
19. Nesvizhskii, A. I., Keller, A., Kolker, E. & Aebersold, R. A statistical model for identifying proteins by tandem mass spectrometry. *Anal. Chem.* **75,** 4646–4658 (2003).
20. Pham, T. V., Piersma, S. R., Warmoes, M. & Jiménez, C. R. On the beta-binomial model for analysis of spectral count data in label-free tandem mass spectrometry-based proteomics. *Bioinformatics* **26,** 363–369 (2010).
21. Yoneyama, M., Kitayama, T., Taniura, H. & Yoneda, Y. Immunohistochemical detection by immersion fixation with Carnoy solution of particular non-N-methyl-D-aspartate receptor subunits in murine hippocampus. *Neurochem. Int.* **44,** 413–422 (2004).
22. Colquhoun, D., Jonas, P. & Sakmann, B. Action of brief pulses of glutamate on AMPA/kainate receptors in patches from different neurones of rat hippocampal slices. *J. Physiol. (Lond.)* **458,** 261–287 (1992).
23. de Kock, C. P. J., Burnashev, N., Lodder, J. C., Mansvelder, H. D. & Brussaard, A. B. NMDA receptors induce somatodendritic secretion in hypothalamic neurones of lactating female rats. *J. Physiol. (Lond.)* **561,** 53–64 (2004).
